# Nanopore Ultra-Long Sequencing for FSHD: From Molecular Diagnosis to Preimplantation Genetic Testing

**DOI:** 10.3390/genes17091104

**Published:** 2026-09-11

**Authors:** Jingjing Li, Yongjie Cheng, Lin Su, Chengyuan Yan, Zhenhua Cao

**Affiliations:** 1 Center of Applied Biotechnology, School of Life Sciences and Technology, Wuhan University of Bioengineering, Wuhan 430415, China; 2 Beijing Bai’an Jimin Medical Technology, Beijing 100080, China

**Keywords:** facioscapulohumeral muscular dystrophy, D4Z4, nanopore sequencing, preimplantation genetic testing, haplotype phasing, DNA methylation, *DUX4*

## Abstract

Facioscapulohumeral muscular dystrophy (FSHD) is a genetically and epigenetically complex autosomal dominant myopathy that presents formidable challenges to molecular diagnosis and reproductive intervention. The disease is caused by aberrant derepression of the *DUX4* retrogene within the D4Z4 macrosatellite repeat array at chromosome 4q35, triggered either by pathological contraction of the array on a permissive 4qA haplotype (FSHD1, ~95% of cases) or by mutations in epigenetic modifier genes *SMCHD1*, *DNMT3B*, and *LRIF1* that lead to global D4Z4 hypomethylation (FSHD2, ~5% of cases). Traditional approaches (Southern blotting, optical genome mapping, bisulfite sequencing) are discontinuous and labor-intensive. Nanopore ultra-long read sequencing spans the entire D4Z4 array in single reads, simultaneously resolving repeat number, haplotype, and allele-specific CpG methylation without bisulfite conversion. With the telomere-to-telomere (T2T-CHM13) reference genome, long-range haplotype phasing enables preimplantation genetic testing for monogenic conditions (PGT-M) for families with de novo pathogenic variants and somatic mosaicism, groups previously excluded from reproductive genetic intervention. This review systematically examines FSHD molecular mechanisms, the Nanopore diagnostic workflow integrated with T2T-CHM13, Nanopore-based PGT-M clinical data, and future perspectives including R11 pore chemistry, AI-driven bioinformatics, CRISPR-targeted enrichment, and multi-omics integration.

## 1. Introduction

Among the inherited myopathies, facioscapulohumeral muscular dystrophy (FSHD; OMIM #158900, #158901) ranks as the third most common, with worldwide estimates placing prevalence between 1:8500 and 1:20,000 [1,2]. A recent nationwide epidemiological survey identified FSHD1 as the single most prevalent adult-onset muscular dystrophy, with clinical severity showing clear genotype–phenotype correlations [3,4]. Symptom onset typically occurs during the second or third decade. What makes FSHD clinically distinctive is its highly asymmetric, descending pattern: weakness starts in the face, then spreads to the shoulder girdle and upper arms, eventually involving the pelvic girdle and lower extremities [5,6,7]. About one in five patients will eventually depend on a wheelchair; as a rule, smaller D4Z4 repeat arrays predict earlier onset and more aggressive disease [8,9].

The molecular event in FSHD is the misexpression of double homeobox 4 (*DUX4*)—a transcription factor encoded as a retrogene within each 3.3 kb unit of the D4Z4 macrosatellite repeat array on chromosome 4q35 [10,11,12,13]. In healthy muscle tissue, this D4Z4 array, which contains 11 to over 150 repeat units, stays locked in a tightly repressed heterochromatic state through dense CpG methylation, H3K9me3 histone marks, and polycomb repressive complexes [14,15,16,17]. The disease state emerges when that repression breaks down. In FSHD1, the breakdown follows physical contraction of the array [11]; in FSHD2, it results from loss-of-function mutations in chromatin regulators—*SMCHD1* being the most frequent, with *DNMT3B* and *LRIF1* contributing less commonly [18,19,20]. Either route, when combined with a permissive 4qA haplotype that supplies a polyadenylation signal for the distal *DUX4* transcript, unleashes toxic DUX4 protein in skeletal muscle [11,12].

*DUX4* functions as a pioneer transcription factor that activates a broad spectrum of germline-specific genes, endogenous retroelements, and immune mediators that are normally silenced in somatic tissues [13,21,22,23]. This aberrant transcriptional program induces oxidative stress, disrupts myogenic differentiation, activates caspase-mediated apoptosis, and impairs muscle regeneration [24,25,26,27,28]. The downstream molecular consequences create a self-perpetuating cycle of muscle degeneration and inflammation that underlies the progressive clinical course of FSHD [29,30,31].

Despite decades of progress in dissecting the molecular basis of FSHD, translating that knowledge into routine clinical diagnostics has proven to be stubbornly difficult. The root problem is structural: conventional short-read next-generation sequencing (NGS) simply cannot resolve the D4Z4 array—it is far too repetitive—nor can it reliably distinguish chromosome 4q35 reads from their 10q26 paralogs, let alone capture the epigenetic modifications that define the disease state [32,33,34]. In practice, a complete diagnostic workflow demands three or more separate technologies: Southern blotting for repeat sizing, SSLP-based or sequence-based haplotyping, and bisulfite sequencing for CpG methylation [35,36]. Each assay requires high-molecular-weight (HMW) DNA, with each running on its own timeline, and integrating the results falls squarely on the clinical interpretation team. For PGT-M, the barriers are even higher. The conventional linkage-based approach needs multi-generation pedigree data that is frequently unavailable, and it cannot directly interrogate D4Z4 from the 5–10 cells obtainable from a single blastocyst biopsy [37,38,39,40].

Oxford Nanopore Technologies (ONT) ultra-long read sequencing attacks each of these diagnostic bottlenecks head-on. Its core advantage lies in ultra-long read lengths: single DNA strands pass through a biological Nanopore, and base sequences are decoded from ionic current traces instead of optical signals or sequencing-by-synthesis readouts. Because there is no inherent length limit beyond the DNA molecule itself, reads routinely exceed 100 Kb, more than enough to cover the entire D4Z4 array in one pass [41,42,43,44,45]. That means D4Z4 repeat units can be counted directly from raw reads rather than inferred from gel migration. At the same time, the raw current signal carrying information about DNA modifications 5-methylcytosine (5mC) can be called at single-molecule resolution without any bisulfite treatment [46,47,48]. And because the reads span the whole subtelomeric region, haplotype phasing across 4q35 becomes a read-level operation, sidestepping the recombination problem that has historically limited linkage-based PGT-M [49,50]. Most importantly, when these ultra-long reads are aligned to the T2T-CHM13 reference genome [33,51,52,53,54]—which resolved every gap, including the full D4Z4 repeat array—analytical precision reaches a level that was simply unattainable with the fragmented GRCh38 assembly.

## 2. Molecular Mechanism of FSHD

### 2.1. The D4Z4 Macrosatellite and DUX4

**Figure 1:**
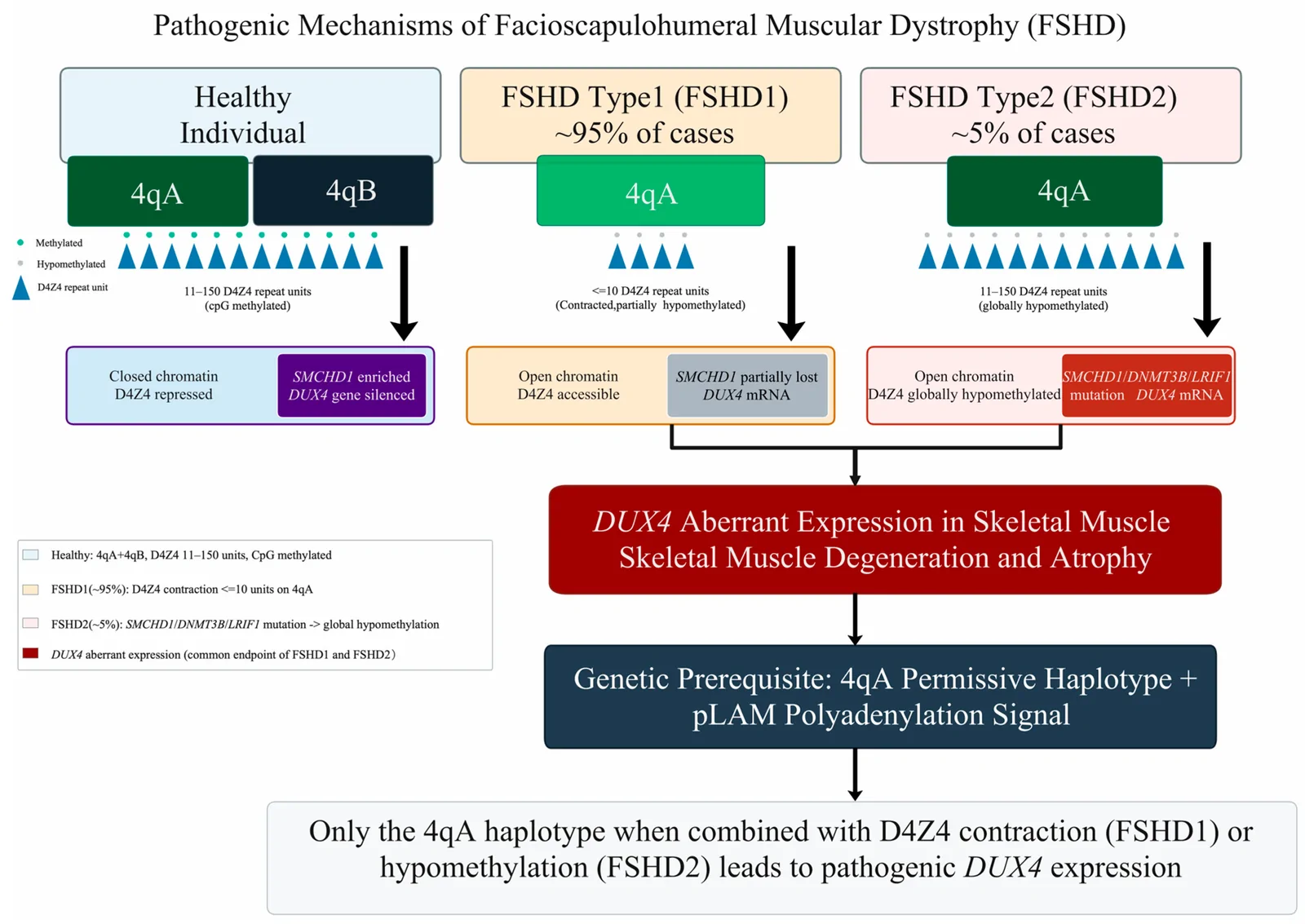
Pathogenic mechanisms and clinical subtypes of FSHD.

At the end of chromosome 4q sits the D4Z4 macrosatellite: a head-to-tail array of 3.3 kb repeat units, each harboring the open reading frame for *DUX4* (Figure 1) [55,56]. The number of repeats in the general population ranges from 11 to more than 150 copies [55,57]. Each D4Z4 unit contains a promoter region, the *DUX4* coding sequence, and regulatory elements embedded within a GC-rich locus [10,56,58]. The 4q35 region exists in two major haplotypic configurations: 4qA, which contains a polyadenylation signal (PAS) distal to the last D4Z4 unit enabling stable *DUX4* mRNA expression, and 4qB, which lacks this PAS and does not produce stable *DUX4* transcripts [11,12]. FSHD1 requires the coincidence of D4Z4 contraction on a 4qA haplotype, representing a unique digenic mechanism in human genetics [59]. The 10q26 subtelomere carries a near-identical D4Z4-like array, and discriminating 4q from 10q reads has been a persistent technical challenge that the long-read era has finally put to rest [32,45]. Importantly, the T2T-CHM13 reference genome has revealed that D4Z4-like repeats are not restricted to the canonical 4q35 and 10q26 arrays; additional loci—several of which retain intact *DUX4* open reading frames or polyadenylation signals—are dispersed across at least ten other chromosomes [60]. These paralogous copies can be co-amplified by primer sets commonly used for *DUX4* or *DBE-T* detection and must be accounted for in the design and interpretation of PCR-based assays [60]. A particularly important nuance is that D4Z4 units are not perfectly identical; single-nucleotide variants scattered through the array create unit-level haplotypes that serve as critical landmarks for phasing [32,45].

### 2.2. DUX4 Transcriptional Program and Muscle Toxicity

*DUX4* is an ancient transcription factor whose normal job is confined to a very narrow developmental window. During zygotic genome activation—the transition from maternal to embryonic transcriptional control—*DUX4* opens chromatin at cleavage-stage genes and mobilizes endogenous retroviral elements, notably MERVL and HERVL families [21,22,23]. When aberrantly expressed in adult skeletal muscle, *DUX4* activates this early developmental program in a somatic context, driving expression of germline-specific genes, immune mediators, and retrotransposons that are normally silenced [13,21,61]. Key downstream targets include *ZSCAN4*, *PRAMEF* family genes, *TRIM43*, and *RFPL* family members [13,21]. *DUX4* expression induces oxidative stress through the generation of double-stranded RNA, stabilizes MYC mRNA, and triggers caspase 3/7-mediated apoptosis [26,28,29]. Furthermore, *DUX4* disrupts myogenic differentiation by interfering with *PAX7* and *MYOD* transcriptional networks [30], and it activates p53-dependent pathways that contribute to muscle degeneration [27,28]. The fact that these pathways reinforce each other—oxidative damage sensitizes cells to apoptosis, dying cells recruit inflammatory infiltrates, and inflammation further compromises regeneration—partly explains why the disease, once established, tends to progress rather than stabilize.

### 2.3. FSHD1: D4Z4 Repeat Contraction

FSHD1 (OMIM #158900) accounts for approximately 95% of all FSHD cases and is caused by contraction of the D4Z4 macrosatellite repeat array at chromosome 4q35 to 1–10 repeat units, occurring exclusively on a permissive 4qA haplotype (Figure 1) [11,55,57]. The contraction removes a critical threshold of D4Z4 units necessary to maintain the repressive heterochromatic state at the locus, resulting in reduced CpG methylation, loss of histone H3K9 trimethylation and HP1γ binding, and chromatin relaxation that permits sporadic *DUX4* transcription in a subset of myonuclei [11,14,15,16]. Disease severity shows an inverse correlation with residual D4Z4 repeat number: patients with 1–3 repeat units (RUs) typically present with earlier onset, more rapid progression, and higher penetrance, while those with 8–10 RUs (borderline alleles) show reduced penetrance (~40% for 8–9 RUs in males) and milder phenotypes [5,8,9]. Approximately 10–30% of FSHD1 cases arise from de novo contractions with no family history, and an estimated 10–20% of patients exhibit somatic mosaicism, carrying a mixture of contracted and non-contracted alleles due to post-zygotic contraction events [59,62].

### 2.4. FSHD2: Epigenetic Modifier Mutations

FSHD2 (OMIM #158901) accounts for the remaining ~5% of patients and illustrates that D4Z4 pathology is fundamentally epigenetic (Figure 1). These individuals carry D4Z4 arrays in the low-normal or borderline range (typically 9–20 units) on a permissive 4qA haplotype, combined with a heterozygous pathogenic variant in a chromatin modifier gene [18,19,20,63]. The most commonly implicated is SMCHD1, a protein that establishes and maintains CpG methylation at the D4Z4 locus during early development [18,20,64,65]. Loss of one functional *SMCHD1* copy leads to marked D4Z4 hypomethylation—functionally equivalent to the chromatin relaxation that occurs in FSHD1 via physical contraction. *DNMT3B* dysfunction contributes to a smaller fraction of FSHD2 cases [19,66,67], and *LRIF1* mutations are even rarer, but they establish the principle that multiple chromatin pathways converge on D4Z4 silencing [63]. A clinically important subset of patients carry both a borderline-short D4Z4 array (8–10 units) and an *SMCHD1* mutation; these “digenic” cases tend to show particularly severe and early-onset disease, suggesting additive or synergistic effects of the two mechanisms [19,67]. The diagnostic implication is clear: any FSHD workup that cannot simultaneously detect *SMCHD1/DNMT3B/LRIF1* variants alongside D4Z4 repeat number and methylation status is, by definition, incomplete [68,69].

## 3. Current Diagnostic Paradigm and Its Limitations

### 3.1. Traditional Diagnostic Workflow

The molecular diagnosis of FSHD has historically required a multi-step, multi-technology workflow that remains the clinical standard in most diagnostic laboratories worldwide (Table 1) [34,35]. The 2024 best practice guidelines for FSHD genetic diagnostics update the 2012 gold standard and specify three essential diagnostic components: (1) determination of the D4Z4 repeat number on chromosome 4q35 using pulsed-field gel electrophoresis and Southern blotting with specific probes (p13E-11, 4qA, 4qB), a procedure requiring 10–15 micrograms of HMW genomic DNA and 2–4 weeks of processing time; (2) 4qA/4qB haplotype determination to confirm the presence of the permissive polyadenylation signal; (3) FSHD2, *SMCHD1*, *DNMT3B*, and *LRIF1* gene sequencing by NGS plus D4Z4 methylation analysis by bisulfite sequencing [18,19,34,63]. This fragmented approach presents significant practical challenges, including high DNA quantity requirements incompatible with small biopsy specimens, prolonged turnaround times of 3–6 weeks, limited availability to specialized reference centers, and the inability to resolve the homologous 10q26 D4Z4-like repeats without complex subtraction protocols [34,35,36].

### 3.2. Optical Genome Mapping and Molecular Combing: Incremental Improvement

Optical genome mapping (OGM) using the Bionano Genomics Saphyr platform has emerged as a validated alternative to Southern blotting for FSHD1 diagnosis [35,36]. OGM fluorescently labels ultra-high-molecular-weight (UHMW) DNA (>150 kb) at specific sequence motifs and images linearized molecules in massively parallel nanochannel arrays, enabling direct visualization and sizing of the D4Z4 repeat array without amplification or electrophoretic separation [35]. Clinical validation by Stence et al. (2021) across 315 clinical samples demonstrated high concordance with Southern blot allele sizing and accurate haplotype assignment [35]. Subsequent molecular diagnosis studies confirmed OGM’s clinical utility and also highlighted its ability to detect complex structural rearrangements at 4q35 [36]. Molecular combing is another single-molecule imaging technique that has been used as an alternative to Southern blotting for FSHD1 diagnosis [72]. This method stretches individual DNA fibers on a silanized glass surface and detects D4Z4 arrays, as well as their flanking regions, via hybridization with fluorescent probes [73]. Molecular combing requires no restriction enzyme digestion, enables direct visualization of individual D4Z4 arrays at high resolution, can detect low-level mosaic contracted alleles that may be missed by Southern blotting, and distinguishes 4q from 10q alleles using locus-specific probes [72,74]. However, molecular combing is labor-intensive and has lower throughput than OGM, and its clinical adoption has remained more limited. However, both OGM and molecular combing share a fundamental limitation: neither can detect CpG methylation status, requiring parallel bisulfite sequencing for complete FSHD2 diagnosis [34,35]. Additionally, neither technique can identify *SMCHD1*, *DNMT3B*, or *LRIF1* mutations, cannot directly phase SNVs for PGT-M haplotype construction, and provides a lower resolution for detecting somatic mosaicism with low-level contracted alleles compared to single-molecule sequencing approaches [32,45]. In brief, while OGM and molecular comb both unify repeat array sizing and paralogous 4q/10q differentiation on a single analytical platform, neither resolves the core issue of disjointed multi-assay diagnostic workflows.

### 3.3. Fundamental Limitations for PGT-M

For couples at risk of transmitting a monogenic disorder, PGT-M is a reproductive technology in which embryos generated by in vitro fertilization are biopsied at the blastocyst stage, which are typically 5–10 trophectoderm cells, and screened for the familial pathogenic variant before being transferred into the uterus [39]. Embryo genotyping generally follows one of the two complementary strategies. When the causative variant can be amplified directly from the few biopsied cells, direct mutation testing is applied. When it cannot—such as for repeat expansions, structural rearrangements, or variants embedded in GC-rich and repetitive regions—PGT-M instead relies on indirect linkage-based haplotype analysis, in which informative polymorphic markers flanking the disease locus are genotyped in the parents and available relatives so that the affected parental chromosome can be traced into each embryo by segregation analysis [39,40]. This is why linkage analysis is central to PGT-M for such disorders: the mutation itself is not measurable from picogram-scale embryonic DNA, and only the segregation of linked markers can be reliably scored. Modern PGT-M workflows are predominantly NGS-based, reconstructing SNP haplotypes from low-coverage whole-genome or targeted sequencing of parental and embryonic DNA, which has largely superseded the microsatellite-based typing of earlier protocols [39]. FSHD represents an extreme case of these constraints, as detailed below.

Traditional FSHD PGT-M approaches face compounding limitations that have restricted clinical success [37,38]. Indirect linkage-based PGT-M requires informative SNP markers flanking the D4Z4 locus, genotyped across multiple generations of affected and unaffected family members [39,40]. This prerequisite is impossible to satisfy in families with de novo mutations, which account for 10–30% of FSHD1 cases, and in small kindreds with limited reference individuals [37,59]. Recombination events between linked markers and the D4Z4 locus, occurring at approximately 5% per meiosis, represent a systematic source of diagnostic error [40,75]. Xu et al. (2026) provided a striking illustration: the last informative SNP identified by Sanger sequencing in their mosaic FSHD1 family was located 224 kb upstream of the D4Z4 locus, creating a recombination gap that rendered three of eight embryos unclassifiable by short-read methods alone [37]. Direct interrogation of the D4Z4 array from embryo biopsies is technically impossible due to the extremely limited DNA quantity from 5–10 trophectoderm cells, requiring whole-genome amplification that introduces dropout and bias at repetitive regions [76,77]. These cumulative constraints historically limited clinical success to approximately 60% and entirely precluded PGT-M in de novo mutation families [37,38].

## 4. The Advantages of Nanopore Ultra-Long Read Sequencing for FSHD

### 4.1. Core Sequencing Principles

**Figure 2:**
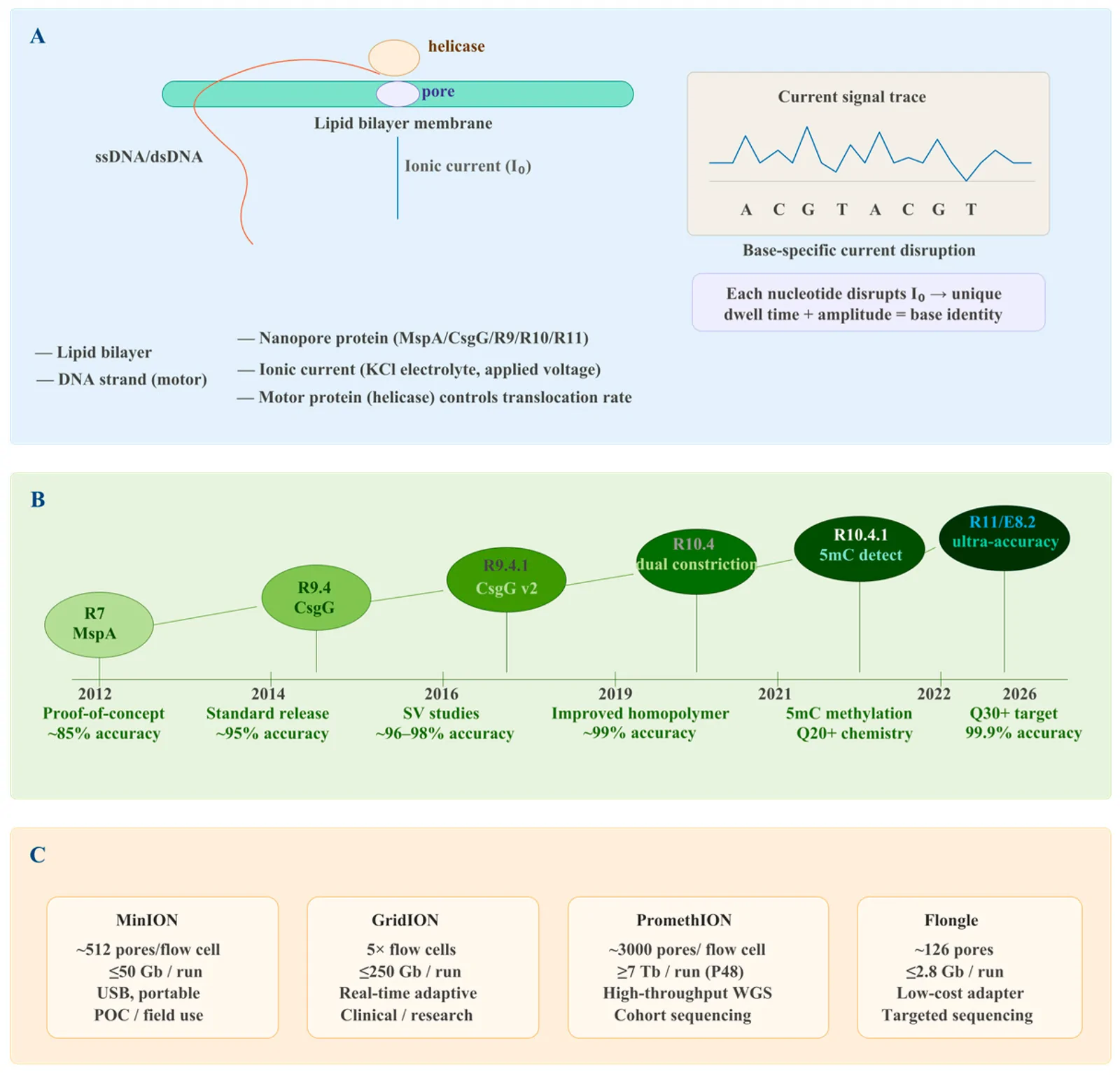
Nanopore sequencing core mechanism and technological development timeline. (**A**) Schematic representation of the Nanopore sequencing principle: single-stranded DNA translocates through a protein Nanopore embedded in a synthetic polymer membrane under an applied voltage, with a motor protein controlling translocation speed. (**B**) Key developmental milestones in ONT technology from the MinION early access program to R11 chemistry. (**C**) Hardware platform comparison: from portable MinION for point-of-care use to high-throughput PromethION P48 for population-scale whole-genome sequencing.

Nanopore sequencing operates on an elegantly simple physical principle. A single protein pore—typically an engineered CsgG from *Escherichia coli*—is inserted into a synthetic polymer membrane separating two electrolyte-filled chambers (Figure 2) [41,78,79]. When a voltage is applied, ions flow through the pore, creating a stable baseline current. A motor protein ratchets a single-stranded DNA or RNA molecule through the pore at a controlled speed, and as each nucleotide passes the narrowest constriction, its chemical identity—purine vs. pyrimidine, and within each class its specific base—modulates the ionic current in a characteristic way (Figure 2A). The continuous current trace is then decoded into a sequence by a neural network basecaller [80,81]. The beauty of this approach is that it sidesteps virtually every limitation of sequencing by synthesis: there is no polymerase, no fluorophore, no clonal amplification, and no inherent read-length ceiling. The current generation of ONT platforms uses the CsgG Nanopore derived from *E. coli* (Table 2), engineered through extensive mutagenesis for optimal signal discrimination [82,83,84]. The R10.4.1 pore chemistry, introduced in 2022, features a dual-constriction design with a longer sensing region that enables Q20+ (99% modal) raw read accuracy and Q30+ consensus accuracy with sufficient coverage (Figure 2B) [44,85,86]. Equally important for FSHD applications, the chemistry now supports N50 read lengths commonly exceeding 100 kb.

### 4.2. Five Key Advantages for FSHD Analysis

#### 4.2.1. Advantage 1: Direct and Single-Molecule Repeat Counting

Because individual Nanopore reads routinely exceed 100 kb, a single read can span the entire D4Z4 array (which at 10 repeats is ~33 kb). This makes it possible to count repeat units directly from raw read alignments, with no amplification bias and no reliance on assembly algorithms [32,45]. When aligned to T2T-CHM13, the D4Z4 repeat-counting precision achieves a coefficient of variation (CV) below 2%, compared to 8–12% with GRCh38-based alignment and entirely unreliable results with short-read methods [33,45]. Perhaps more importantly, mosaic genotypes—where different cells carry different repeat arrays—can be detected and quantified because each read represents an independent single-molecule observation.

#### 4.2.2. Advantage 2: Simultaneous Methylation Detection

Here is what sets Nanopore apart from other sequencing platforms for FSHD: the raw electrical signal—not the basecalled sequence—carries methylation information. When a 5mC passes through the pore, it produces a subtly different current perturbation than unmethylated cytosine, and these differences can be detected at single-nucleotide, single-molecule resolution [46,47,48]. For the D4Z4 locus, this means you can quantify methylation frequency at individual CpG sites and—critically—call methylation separately on each allele. Tools like Nanopolish, Megalodon, and Dorado with the remora modification-calling module have made this practical in routine analysis workflows [47,87,88]. The ability to simultaneously read sequences and methylation from the same molecule collapses what used to be a two-assay workflow (sequencing + bisulfite) into one. And because there is no bisulfite conversion, there is no C-to-T bias, no degradation of input DNA, and no ambiguity about incomplete conversion rates.

#### 4.2.3. Advantage 3: Allele-Specific Discrimination of Chromosome 4q and 10q

One of the most persistent artifacts plaguing FSHD genetic analysis has been the misassignment of reads between the nearly identical D4Z4 arrays on chromosomes 4q35 and 10q26. With short reads (100–300 bp), more than 20% of 4q-derived reads misalign to 10q coordinates in GRCh38, and vice versa [33]. Nanopore ultra-long reads solve this problem decisively: flanking unique sequences on either side of the repeat array unambiguously identify the chromosome of origin [32,45]. With T2T-CHM13 as the reference, which correctly assembles both the 4q35 and 10q26 D4Z4 arrays, the discrimination is near-perfect.

#### 4.2.4. Advantage 4: Concurrent Genome-Wide Detection of FSHD2 Genes

A single Nanopore whole-genome sequencing run interrogates *SMCHD1*, *DNMT3B*, and *LRIF1* alongside the D4Z4 locus, providing comprehensive FSHD1/FSHD2 differential diagnosis without additional targeted panels or separate sequencing runs [18,19,45,63]. The long reads are particularly valuable for *SMCHD1*, which spans ~200 kb on chromosome 18p and contains several highly repetitive exons that are poorly covered by short-read sequencing. Structural variant large deletions, duplications, and inversions that would be invisible to short-read panels can be detected directly from read-level alignment patterns [18,89]. For FSHD2, the diagnostic requirement differs fundamentally and exposes the limits of both alternative platforms. A complete FSHD2 workup must achieve two things simultaneously: (1) detect pathogenic sequence variants in *SMCHD1*, *DNMT3B*, and *LRIF1* including large structural rearrangements and deep intronic changes that are frequently missed by targeted short-read panels [89]; (2) demonstrate D4Z4 hypomethylation to establish the epigenetic diagnosis [34]. Short-read NGS is well suited to exonic SNVs and small indels in the FSHD2 genes, but it cannot resolve large *SMCHD1* deletions, duplications, and inversions or the full intronic architecture of the locus, and it provides no methylation information without a parallel bisulfite-based assay [34,89]. OGM, in turn, offers excellent structural resolution of the 4q35 repeat array but yields no sequence and no methylation signal, so an FSHD2 diagnosis by OGM still requires additional gene sequencing and bisulfite testing [34,35,36]. Nanopore whole-genome sequencing can fulfill both requirements in a single assay, combining read-level detection of *SMCHD1* structural and intronic variants with single-molecule, allele-specific D4Z4 methylation calling from the same ultra-long reads [18,46,47,63,89].

#### 4.2.5. Advantage 5: Direct Haplotype Phasing for PGT-M

Ultra-long reads that span the entire 4q35 subtelomere provide read-level haplotype information, phasing informative SNPs directly onto the D4Z4 disease allele without needing any pedigree data [37]. This is a game changer for PGT-M because it eliminates the recombination error inherent in linkage-based approaches and makes PGT-M feasible for de novo mutation families—the very population that has historically been most in need of reproductive options and least able to access them [49,50]. Block-based phasing across hundreds of kilobases also provides dense flanking markers, reducing the probability that a recombination event goes undetected even further compared with sparse SNP-based linkage.

## 5. Nanopore-Based FSHD Diagnostic Workflow

### 5.1. D4Z4 Repeat Sizing and Haplotype Determination

**Figure 3:**
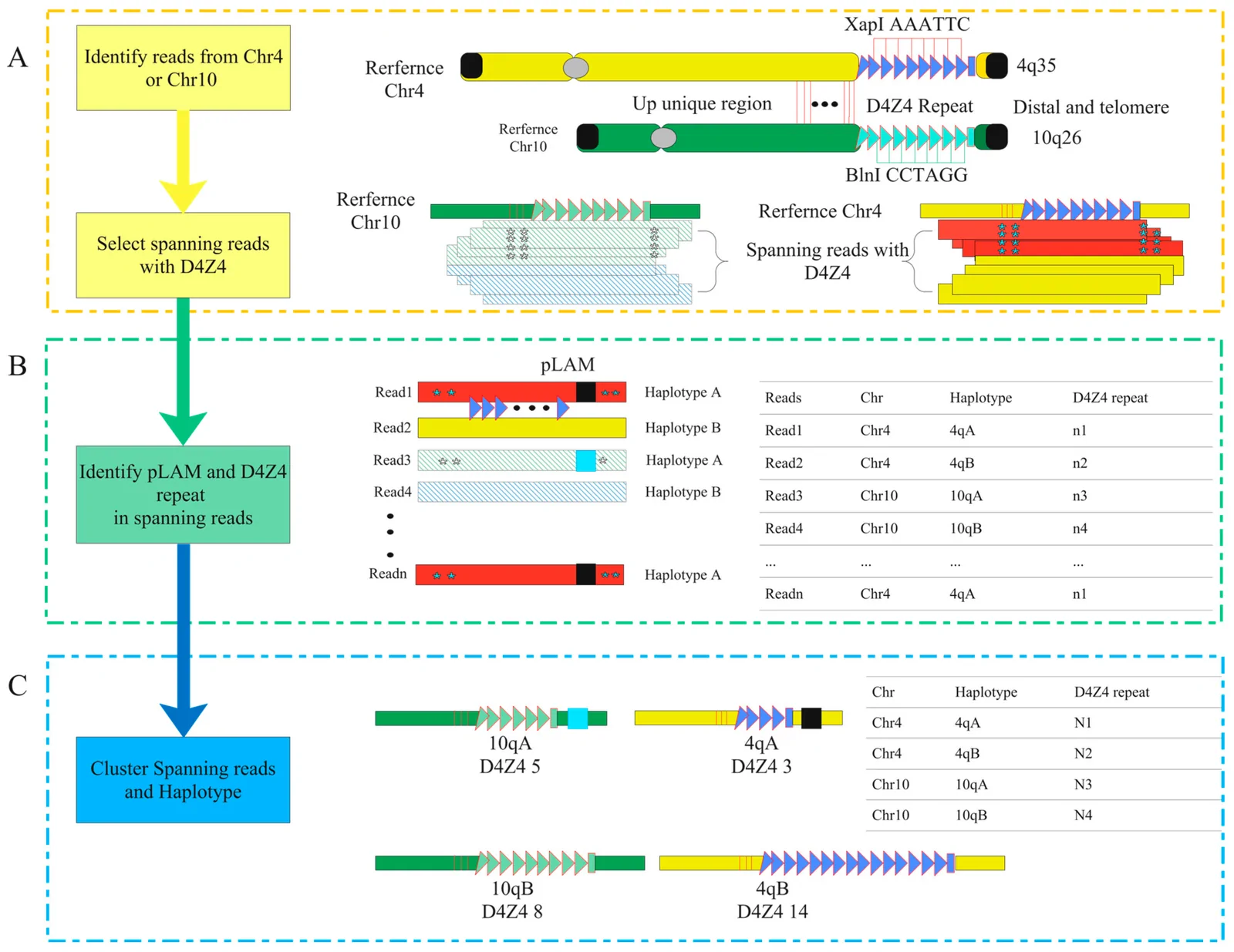
Nanopore ultra-long read sequencing for FSHD diagnosis workflow. (**A**) Classify reads originating from chr4 or chr10, and filter ultra-long reads that fully span the D4Z4 repeat array. (**B**) Detect pLAM haplotype markers and enumerate D4Z4 repeat units in each spanning read, annotating each read with chromosome, haplotype, and repeat copy number. (**C**) Cluster spanning reads by chromosome and haplotype to resolve complete D4Z4 arrays and obtain precise repeat counts for each haplotype.

The Nanopore-based FSHD diagnostic workflow achieves comprehensive molecular characterization in a unified assay (Figure 3) [45,71]. Huang et al. (2024) validated an ONT WGS-based FSHD detection method in a case–control design of 29 samples, comparing results with OGM, BSS, and WES (Table 1) [45]. The method accurately determined D4Z4 repeat number, 4qA/4qB haplotype, and allele-specific CpG methylation at the most distal D4Z4 unit. Butterfield et al. (2023) combined Nanopore long-read sequencing with Cas9-targeted enrichment of 4q and 10q D4Z4 arrays, revealing asymmetric, length-dependent methylation gradients that reach a hypermethylation plateau at approximately 10 D4Z4 repeat units [32]. The recently developed DUCKS4 comprehensive workflow integrates optimized wet-lab protocols with automated bioinformatic analysis for Nanopore-based FSHD diagnosis, further standardizing the pipeline for clinical implementation [71]. Xiao et al. (2026) developed D4Z4End2End, which combines ultra-long whole-genome sequencing with Cas9-targeted enrichment for haplotype-resolved reconstruction and methylation profiling of complete D4Z4 arrays, accurately capturing contracted alleles, genetic mosaicism, and pathogenic *SMCHD1* variants in FSHD1 and FSHD2 cohorts [90]. As reported in the literature, the core analytical pipeline involves: (1) library preparation for HMW genomic DNA using the ONT ligation sequencing kit; (2) sequencing on a PromethION flow cell (generating 20–30× coverage, >50 Gb); (3) basecalling with Dorado using the super-accuracy (sup) model; (4) alignment to T2T-CHM13 with minimap2 using parameters tuned for ultra-long reads; (5) D4Z4 repeat quantification and 4qA haplotype characterization at the 4q35 locus [32,45,71].

### 5.2. Allele-Specific Epigenetic Profiling

Nanopore sequencing uniquely enables allele-specific DNA methylation profiling at single-molecule resolution [46,47]. At the D4Z4 locus, methylation frequency at individual CpG sites can be quantified separately for the 4qA and 4qB alleles, the pathogenic contracted allele versus the non-contracted allele, and the 4q35 versus 10q26 D4Z4 arrays [32,45]. This resolution reveals disease-relevant epigenetic heterogeneity that is completely masked by population-average bisulfite sequencing. Sharim et al. (2019) demonstrated that long-read single-molecule maps can resolve the functional methylome at repetitive regions, providing a technical framework applicable to FSHD [87]. DeepSignal and comparable deep learning methylation callers reach > 95% correlation with bisulfite sequencing at single CpGs, with allele-specific resolution unavailable to BSS [48,88].

Moving from allele-level to position-level resolution explains why methylation position matters for the transcriptional outcome. Methylation is not uniform across the D4Z4 array: asymmetric, length-dependent methylation gradients have been mapped across the 4q and 10q arrays, reaching a hypermethylation plateau at approximately 10 repeat units [32]. Within this architecture, the CpG positions in and around the *DUX4* promoter and polyadenylation signal of the most distal repeat unit are considered the most functionally consequential, because hypomethylation at these distal positions is thought to gate productive *DUX4* transcription [11,12], whereas methylation changes across the internal units modulate the overall repressive chromatin state of the array without individually determining the *DUX4* output. Population-average bisulfite sequencing collapses all of this positional information into a single array-wide value and therefore cannot distinguish functionally relevant distal hypomethylation from biologically neutral internal variation. Single-molecule long-read methylation calling preserves position, molecule, and allele simultaneously, providing exactly the positional resolution needed to link methylation at individual CpG sites to transcriptional activation or repression of *DUX4* [32,46,47,87].

Beyond 5-methylcytosine, Nanopore basecalling frameworks can be retrained to distinguish 5-hydroxymethylcytosine, 4-methylcytosine, and N6-methyladenine [46,48]. Histone acetylation, by contrast, is a protein-level modification that cannot be read directly from the DNA strand; chromatin-state information relevant to *DUX4* regulation therefore still requires complementary assays such as ChIP-seq or CUT&Tag approaches that can be directly informed by the allele- and position-resolved methylation maps that Nanopore sequencing provides [87].

### 5.3. Concurrent Genome-Wide Detection of FSHD2 Genes

A key advantage of the Nanopore WGS approach is the concurrent detection of *SMCHD1*, *DNMT3B*, and *LRIF1* variants in the same sequencing run [18,19,45,63]. Long reads spanning these genes can detect not only single-nucleotide variants and small indels but also structural variants—including large deletions, duplications, and inversions that are common in *SMCHD1* and frequently missed by short-read sequencing [89]. Deep intronic *SMCHD1* variants, which account for a significant fraction of genetically unresolved FSHD2 cases, are also detectable through long-read sequencing of the full gene locus [89].

### 5.4. Analytical Synergy with the T2T-CHM13 Reference Genome

The analytical performance of Nanopore-based FSHD diagnostics is substantially enhanced by alignment to the gapless T2T-CHM13 reference genome [33]. The CHM13 assembly fully resolves all repetitive regions that are collapsed or misassembled in GRCh38, including the complete D4Z4 macrosatellite array at 4q35 and the paralogous array at 10q26 [33,51,52,54]. This improvement is critical for FSHD analysis because the 4q35 subtelomeric region remains one of the most poorly assembled regions in GRCh38 due to its repetitive nature [33,91]. Alignment to T2T-CHM13 eliminates the >20% D4Z4 read misalignment rate observed with GRCh38, improves SNV phasing accuracy in the 4q35 flanking region from 82% (GRCh38) to 99.9%, and enables detection of rare structural variations in the 4q35 subtelomeric region associated with atypically severe FSHD phenotypes [33,51,52,92]. The result is that D4Z4 repeat-counting precision approaches the theoretical limit set by read coverage, and 4q versus 10q discrimination becomes a solved problem rather than a source of error [70,93,94].

## 6. Nanopore-Based Preimplantation Genetic Testing for FSHD

### 6.1. Limitations of Conventional PGT-M for FSHD

As described in Section 3.3, PGT-M is a reproductive option in which embryos generated through in vitro fertilization are genetically screened before transfer, either by direct mutation detection or by indirect linkage-based haplotype analysis. Yet FSHD has until recently been a conspicuous gap in the PGT-M portfolio. The underlying reasons are straightforward: direct mutation detection is impossible for a repeat array, while indirect linkage analysis requires a cooperative pedigree and informative markers flanking the disease locus—for FSHD, neither condition is reliably met [39,95,96,97].

Conventional PGT-M approaches have prominent limitations when performing genetic testing for FSHD, as detailed below. First, linkage mapping demands DNA from multiple relatives—affected and unaffected—to phase SNP markers around the D4Z4 locus. In the not-uncommon scenario of a de novo FSHD1 mutation, or when grandparents are deceased, no informative linkage phase can be established. Second, even when a complete pedigree is available, the recombination rate in the subtelomeric 4q35 region is not negligible: a crossover between the marker SNPs and the D4Z4 array occurs in roughly 5% of meioses [37,39,75]. A linkage-based diagnosis that is wrong 5% of the time is a hard sell in a clinical setting where the outcome is embryo selection. Third, and most fundamentally, the 5–10 cells obtained from a trophectoderm biopsy yield perhaps 30–60 pg of genomic DNA—an amount that is utterly insufficient for Southern blotting, OGM, or any method requiring direct visualization of the D4Z4 array. Short-read NGS after whole-genome amplification can provide SNP genotypes [76,77,98], but it cannot reconstruct the repeat structure. These are not minor inconveniences; they are fundamental barriers.

### 6.2. The Nanopore-Based PGT-M Workflow

**Figure 4:**
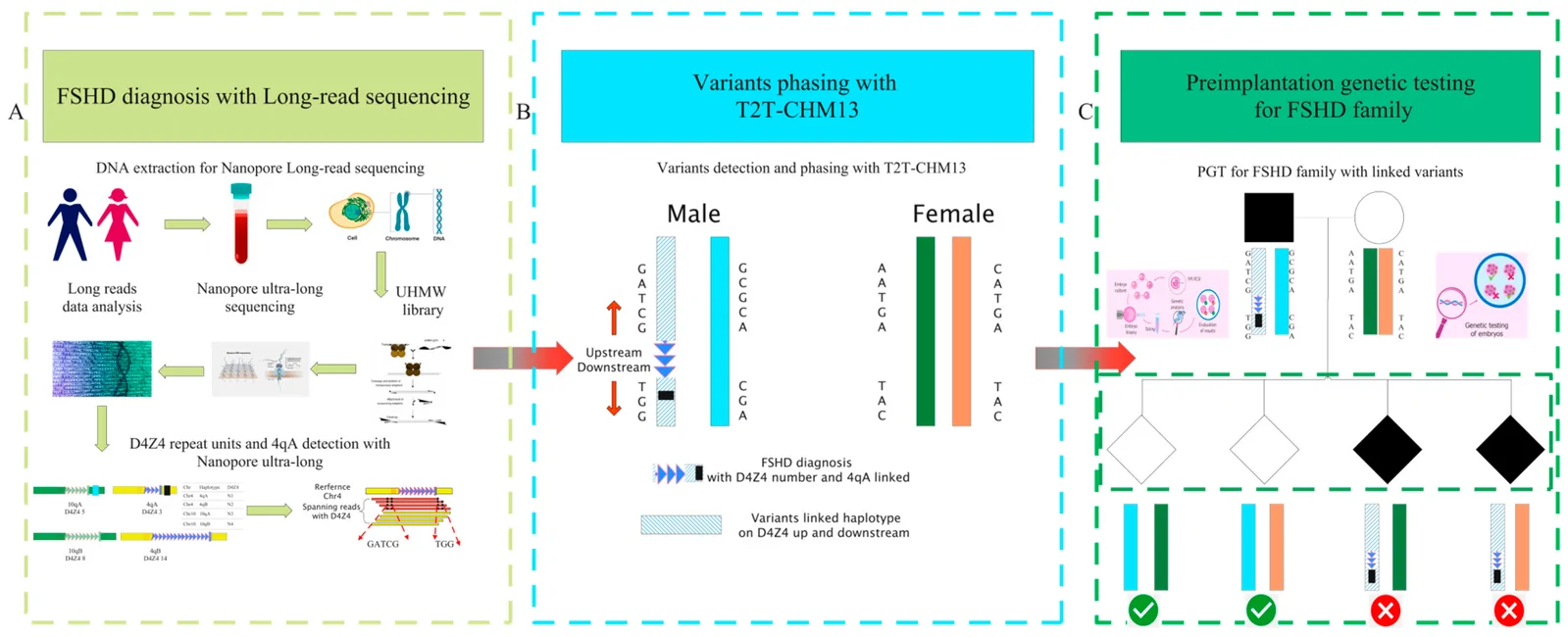
Nanopore ultra-long read sequencing for the FSHD PGT-M workflow. (**A**) FSHD diagnostic workflow based on Nanopore ultra-long read sequencing: including UHMW DNA library preparation, ultra-long read sequencing, and single-molecule resolution of D4Z4 repeat counts and 4qA haplotypes. (**B**) Haplotype phasing of D4Z4-flanking variants using the T2T-CHM13 reference genome to distinguish disease-linked and normal allelic backgrounds. (**C**) Application of phased haplotype markers in FSHD preimplantation genetic testing, for pathogenic haplotype tracing and embryonic risk stratification.

Nanopore ultra-long read sequencing, when combined with T2T-CHM13 alignment and block-based haplotype phasing, systematically dismantles each of the three barriers outlined above (Figure 4). The clinically validated workflow proceeds in four phases, as described below. The key principle throughout is that the ultra-long reads serve a dual purpose: they directly interrogate the D4Z4 locus (for repeat count, haplotype, and methylation) and simultaneously phase-flanking SNPs into disease-linked and wild-type haplotypes—all from a single affected parent DNA sample [37,38,50]. In this workflow, the proband is the affected parent whose D4Z4 haplotype is characterized by ultra-long read sequencing, whereas the embryos are the individuals under preimplantation genetic testing. Sequencing is therefore performed on two distinct sample types for two distinct purposes: long-read whole-genome sequencing is applied to parental blood DNA to establish the disease-linked haplotype, whereas embryo biopsies are not subjected to long-read sequencing but are genotyped after whole-genome amplification using short-read or Sanger sequencing, as described in Phase 3 (Figure 4C).

#### 6.2.1. Phase 1—Pathogenic Haplotype Characterization

ONT ultra-long read WGS (read N50 > 60 kb) is performed on peripheral blood genomic DNA from the affected parent to (Figure 4A): (a) enumerate D4Z4 repeat units and confirm the 4qA haplotype (FSHD1) or identify *SMCHD1*/*DNMT3B*/*LRIF1* mutations with concurrent D4Z4 hypomethylation (FSHD2); (b) phase informative SNVs and indels in the 50–100 kb flanking region of the D4Z4 array using T2T-CHM13-anchored read-level phasing [33,49]; (c) validate the Mendelian heritability and chromosomal stability of phased haplotype markers against parental and sibling genotypes where available [37]. Xu et al. (2026) demonstrated that block-based analysis—connecting heterozygous SNPs across the entire target region into a contiguous phasing block—enables robust haplotype assignment even when downstream informative SNPs are sparse due to the proximity to the telomere [37].

#### 6.2.2. Phase 2—Haplotype Marker Validation and Bioinformatics

Phased SNVs identified in Phase 1 are validated through orthogonal methods including short-read NGS of all available family members, Sanger sequencing of critical recombination-flanking markers, and OGM for structural conformation of the 4q35 region [37,38]. The validated haplotype markers are stored as a curated database for subsequent embryo screening (Figure 4B).

#### 6.2.3. Phase 3—Whole-Genome Amplification and Targeted Embryo Screening

Whole-genome amplification (WGA) by multiple displacement amplification (MDA) increases embryonic genomic DNA from picogram quantities to microgram quantities sufficient for downstream genotyping [76,77]. The amplified DNA is then followed by targeted short-read NGS or Sanger validation. Xu et al. (2026) introduced a critical innovation: when short-read NGS identifies a last informative SNP located at a significant distance from the D4Z4 locus, Nanopore-identified SNPs within this gap region are verified by Sanger sequencing in embryos, effectively closing the recombination gap and enabling definitive embryo classification (Figure 4C) [37].

#### 6.2.4. Phase 4—Embryo Selection and Prenatal Confirmation

Embryos classified as unaffected by haplotype analysis are prioritized for uterine transfer. Prenatal diagnosis via OGM at approximately 17 weeks of gestation provides independent confirmation of D4Z4 repeat number, as demonstrated in both the Xu et al. (2026) and Hu et al. (2025) studies [37,38].

### 6.3. Clinical Validation Data

To date, three independent clinical reports have validated the feasibility of PGT-M for FSHD (Table 3). Xu et al. (2026) applied block-based Nanopore haplotype phasing for PGT-M in a somatic mosaic FSHD1 family at the First Affiliated Hospital of Sun Yat-sen University, Guangzhou, China [37]. The male affected parent carried three distinct D4Z4 haplotypes (4, 16, and 33 RUs) consistent with post-zygotic somatic contraction. Critically, both the pathogenic 4 RUs allele and the non-pathogenic 16 RUs allele originated from the same parental haplotype, dictating that PGT-M must be based on haplotype tracking rather than direct repeat counting. Of eight biopsied blastocysts, five were classified as unaffected by combined three-tier analysis (Nanopore + short-read NGS + Sanger sequencing). Transfer of embryo E1 resulted in a healthy male live birth, with OGM-based prenatal confirmation at 17 weeks gestation [37].

Hu et al. (2025) reported the first successful PGT-M program for FSHD1 at CITIC-Xiangya Hospital, demonstrating SNP-based linkage analysis across six FSHD1 families [38]. A total of 34 blastocysts were biopsied and tested; 18 embryos (52.9%) were classified as unaffected. Four healthy live births were achieved with Bionano OGM prenatal validation. Shani et al. (2023) reported the largest FSHD PGT-M case series at the European Society of Human Genetics conference, presenting outcomes for 21 women from FSHD families who underwent STR-based indirect linkage PGT-M, resulting in 17 healthy live births [99]. While their method still requires pedigree-based linkage and cannot resolve de novo mutations, the study established important benchmarks for clinical PGT-M success rates and demonstrated that embryologists and genetic counselors can integrate FSHD screening into existing IVF workflows.

### 6.4. Ethical and Counseling Considerations

The implementation of PGT-M for FSHD raises important ethical considerations that warrant careful deliberation in genetic counseling practice [100,101]. Unlike many monogenic disorders with predictable, severe early-onset phenotypes, FSHD exhibits highly variable expressivity: approximately one-third of D4Z4 contraction carriers remain asymptomatic throughout life, and even among affected individuals, the age of onset, rate of progression, and ultimate disability are difficult to predict [5,8,68]. This variable expressivity presents a fundamental challenge for embryo selection decisions, as a transferred embryo carrying a non-pathogenic haplotype could theoretically still be at risk for other genomic variants [100,101]. The principle of reproductive autonomy must be balanced against concerns about selecting against embryos with mutations that may have variable or late-onset phenotypes. Non-invasive prenatal testing (NIPT) for monogenic disorders, based on cell-free fetal DNA analysis using relative haplotype dosage or relative mutation dosage approaches, represents a potential future alternative that would avoid the invasive nature and costs of IVF-PGT-M, though its application to FSHD requires further technical development [102].

## 7. Challenges and Future Perspectives

### 7.1. Current Technical Limitations

Practical limitations still constrain the routine clinical deployment of Nanopore-based FSHD diagnostics. Closely related to library quality is the amount and integrity of the starting material, which varies widely across clinical scenarios—and it is here that some of the practical limits of Nanopore sequencing become apparent. Raw read accuracy, while dramatically improved with R10.4.1 and R11 chemistry, remains below that of Illumina short reads; for most FSHD applications (repeat counting, methylation calling, SNP phasing), this is perfectly adequate, but detecting rare subclonal variants in *SMCHD1* or resolving single-nucleotide differences between nearly identical D4Z4 units still benefits from orthogonal short-read confirmation [85,86]. Library preparation imposes a further constraint: the ligation sequencing kit demands 1–2 µg of high-quality HMW DNA with fragments > 50 kb. Peripheral blood provides ample input, but the samples most relevant to reproductive genetics are precisely the smallest: a trophectoderm biopsy yields only 5–10 cells, and amniotic-fluid or chorionic-villus samples are similarly limited—far below the kit’s requirement. The workflow therefore assigns sequencing tasks by scenario: parental haplotyping is always performed on native HMW DNA from blood, whereas embryonic genotyping relies on MDA-based whole-genome amplification, which introduces dropout and bias preferentially at repetitive regions [76,77], restricting embryo-side analysis to targeted assays rather than direct D4Z4 interrogation.

Residual protein, heme, or ethanol from imperfect DNA isolation impairs adapter ligation and promotes pore blocking, degrading throughput; fragmented or contaminated samples show characteristic drops in the read-length distribution (N50) that serve as practical acceptance criteria before a run. Degraded DNA below ~30 kb cannot span the D4Z4 array in a single read. DNA extracted from frozen or formalin-fixed tissue, or from samples that have undergone freeze–thaw cycles, often yields fragments below this threshold—forcing reliance on assembly-based or consensus approaches with reduced single-molecule certainty [103].

Computational infrastructure is the third practical hurdle: a 40× PromethION run generates roughly 2 TB of raw signal data (POD5) plus ~120 GB of basecalled FASTQ, and processing, aligning, and analyzing this volume requires a high-performance computing environment with GPU-accelerated basecalling—an investment that many diagnostic laboratories are not yet equipped to make [44,104,105,106,107,108,109,110]. In practice, high-quality HMW-DNA extraction, cold-chain transport, and routine DNA quality assessment by pulsed-field or capillary electrophoresis prior to library preparation constitute the minimum quality-control standard for clinically reliable Nanopore FSHD diagnostics.

### 7.2. Emerging Technological Developments

Several converging innovations promise to further enhance the clinical utility of Nanopore sequencing for FSHD in the near future. (1) R11 pore chemistry: The next-generation pore design is anticipated to achieve raw read accuracy exceeding Q30 (99.9%), which would enable single-molecule SNV calling without consensus depth requirements and further improve methylation detection sensitivity [41,85]. (2) AI-driven bioinformatics: Deep learning-based tools for basecalling (Dorado), methylation calling (DeepSignal, MicroMethyl), structural variant detection (Sniffles2, cuteSV), and integrated clinical reporting are evolving rapidly and promise to reduce the computational expertise barrier to clinical adoption [48,88,106,108]. (3) CRISPR-based targeted enrichment: Cas9-targeted enrichment combined with adaptive sampling enables selective sequencing of the 4q35 region without whole-genome coverage requirements, potentially reducing sequencing costs by >90% and turnaround times to under 8 h [109,110,111,112]. This approach is particularly attractive for PGT-M applications where targeted analysis of the D4Z4 region is sufficient [109,111]. (4) Multi-omics integration: The combination of genomic, epigenomic, and transcriptomic analysis in a unified long-read platform offers the prospect of comprehensive molecular profiling that captures all layers of FSHD pathology in a single experiment [113,114,115,116]. (5) Direct RNA sequencing: the same single-molecule principle extends to RNA, as native transcripts can be read without reverse transcription or amplification, resolving modifications such as N6-methyladenosine (m6A) that influence RNA stability, splicing, and translational efficiency [117]. For FSHD, this capability is relevant in two respects: *DUX4* transcript processing and stability set the intracellular dose of the toxic gene product, and the antisense oligonucleotide and RNA-interference therapeutics discussed in Section 7.4 act at the RNA level, so transcript-resolved modification and stability profiling may eventually serve as a pharmacodynamic readout for *DUX4*-targeted interventions.

### 7.3. Affordability and Global Accessibility

Affordability and global accessibility represent the final piece of this translation puzzle. As summarized in Table 1, a Nanopore whole-genome assay costs approximately $1000–2000 per sample—roughly half the cost of the combined classical workflow—while the capital cost of entry has fallen to roughly $1000 for a MinION running from a laptop [42,45]. Targeted enrichment and adaptive sampling are expected to reduce per-sample costs by a further order of magnitude [109,110,111,112]. These characteristics are particularly consequential for low- and middle-income countries (LMICs), where FSHD remains underdiagnosed and genetic services are concentrated in a few centers: a single portable sequencer can substitute for the entire three-assay classical workflow, and the remaining barriers to HMW DNA transport, bioinformatics training, and quality management are logistical rather than technological, and are precisely the gaps that the standardization efforts outlined in Section 7.4 are intended to close. Wider adoption in LMICs would therefore translate directly into reduced global diagnostic inequity in neuromuscular genetics. These cost considerations should not overshadow the value of this application. For FSHD families, accurate molecular diagnosis and access to PGT-M enable informed reproductive decisions. Cost reductions should amplify this benefit, not overshadow it.

### 7.4. Toward Clinical Standardization

The transition of Nanopore FSHD diagnostics from research to clinical practice requires coordinated efforts across multiple fronts: the development of standardized operating procedures (SOPs) for library preparation, sequencing, and analysis; the creation of publicly available benchmarking datasets with ground-truth annotations; the establishment of proficiency testing programs through organizations such as the European Molecular Genetics Quality Network (EMQN) and the College of American Pathologists (CAP); and economic modeling to demonstrate cost-effectiveness compared to current multi-technology diagnostic workflows [33,34]. To date, Nanopore-based assays for FSHD diagnosis remain research-use-only (RUO), and no Nanopore-based FSHD diagnostic test has yet received formal regulatory approval for clinical use in any country. However, recent guideline updates and ongoing validation studies indicate that long-read approaches are approaching clinical implementation, and the standardization efforts outlined above will be instrumental in supporting regulatory approval and accredited laboratory adoption. These advances coincide with an evolving therapeutic landscape: although losmapimod—a p38 MAPK inhibitor previously evaluated in the phase 2 ReDUX4 trial—was discontinued for FSHD [118,119,120], other *DUX4*-directed approaches, including antisense oligonucleotides [121,122], RNA interference [123], and BET bromodomain inhibitors, remain in active development and will require precise genotype-stratified patient selection for clinical trials and eventual therapeutic administration [124,125,126,127,128,129,130,131]. The combination of improved diagnostic capabilities and emerging disease-modifying therapies positions FSHD at the threshold of a new era of precision medicine [132,133,134,135,136].

## 8. Conclusions

What we have tried to convey in this review is that Nanopore ultra-long read sequencing does not merely improve FSHD diagnostics incrementally—it fundamentally rewrites what is possible. In a single experiment, one can now count every D4Z4 repeat unit directly from individual DNA molecules, determine the 4qA/4qB haplotype with near certainty, measure allele-specific CpG methylation across the entire array, and screen the three FSHD2 genes for pathogenic variants—all within 48 to 72 h. The alignment to the T2T-CHM13 reference genome eliminates the systematic mapping errors that have hobbled short read-based analyses for years and pushes D4Z4 repeat-counting precision to a coefficient of variation below 2%. The diagnostic logic validated for FSHD provides a common framework for other repeat-mediated disorders. Huntington disease, caused by a CAG repeat expansion in *HTT*, is the paradigm, together with other short-tandem repeat expansion disorders and other complex diseases. For each, Nanopore sequencing provides what conventional workflows cannot: single-molecule repeat counting, interruption detection, complex structural variant detection, and haplotype context from the same molecule. For a field that has spent decades piecing together partial answers from multiple separate assays, this is not just progress—it is a different category of diagnostic capability.

The reproductive genetics dimension may prove to be the most impactful application of all. Nanopore-based PGT-M has now been validated in independent clinical studies and offers three advantages that linkage-based methods simply cannot match: it works without pedigree data, making it applicable to de novo and mosaic cases; it eliminates the ~5% per-meiosis recombination error; and it provides direct haplotype phasing across megabase-scale regions, reducing the risk of undetected crossover events. The published case series—Xu et al. (2026) for mosaic FSHD1, Hu et al. (2025) for familial FSHD1, and Shani et al. (2023) for the largest case series to date—collectively demonstrate that healthy live births following PGT-M are achievable with prenatal OGM confirmation [37,38,99]. Larger prospective cohorts with long-term pediatric follow-up are needed, but the feasibility is no longer in question.

Looking forward, this is a field in motion. R11 pore chemistry pushes raw accuracy beyond Q30; AI-driven basecalling, variant detection, and methylation analysis continue to improve month by month; CRISPR-based targeted enrichment promises to slash the cost and time of FSHD-specific sequencing; and multi-omics integration—direct RNA-seq, single-cell epigenomics, proteomics—will add layers of functional information that go far beyond genotype alone. None of this is happenstance; it reflects the unique convergence of long-read sequencing chemistry, a completed human reference genome, and mature computational methods that together unlock the most challenging region of the human genome for the first time. We believe the evidence now supports a clear clinical recommendation: Nanopore ultra-long read sequencing, anchored to the T2T-CHM13 reference genome, should be adopted as the first-line diagnostic tool for comprehensive FSHD management—covering everything from initial molecular confirmation through reproductive counseling to genotype-stratified therapeutic decision-making.

## Figures and Tables

**Table 1:** Comparison of diagnostic methods for FSHD.

Feature	Nanopore WGS	Bionano	Southern Blot	NGS + BSS	Combined Classical
D4Z4 Repeat Counting	Direct single-molecule counting; CV < 2% with T2T-CHM13 [32,33,45]	Single-molecule optical mapping; CV ~3–5% [35,36]	Gold standard; CV 5–10%; labor-intensive [5,34]	Indirect inference; not reliable [34]	Requires 2+ independent methods
4qA or 4qB Haplotype	Direct read-level phasing; 99.9% accuracy [33,45]	Single-molecule detection [35]	SSLP-based; limited resolution	Short-read SNV phasing [33]	SSLP + Southern blot
CpG Methylation	Single-molecule 5mC detection; allele-specific [32,46,47]	Not detected	Not detected	BSS gold standard; population average [14,15,70]	BSS only
FSHD2 Detection	Concurrent WGS; *SMCHD1*/*DNMT3B*/*LRIF1* [18,19,63]	Not detected	Not detected	Panel/Exome/WGS	Additional NGS
10q26 Discrimination	Direct mapping to 4q/10q [32,45]	Direct mapping to 4q/10q	Requires specific probes; complex	misalignment > 20% [33]	Often unresolved
Turnaround Time	48–72 h [45,71]	3–5 days [35]	2–4 weeks [34]	1–2 weeks [34]	4–6 weeks
Cost per Sample	~$1000–2000 [45]	~$500–800 [35]	~$300–500	~$800–1500	$2000–4000
PGT-M Compatibility	Direct haplotype phasing; block-based [37,38]	Not applicable	Not applicable	SNP linkage; pedigree-dependent [38]	Pedigree-dependent only
De novo Mutation	Direct; no pedigree needed [37]	Possible; limited	Possible; limited	Not possible	Not possible

**Table 2:** Summary of Oxford Nanopore sequencing pore chemistry evolution.

Feature	R7	R9.4	R9.4.1	R10.4	R10.4.1	R11
Release year	2012–2013	2016	2018	2021	2022	2024–2025
Pore protein	MspA [78]	CsgG	CsgG	CsgG R10 [81]	CsgG R10	Novel engineered pore
Pore constriction	Single	Single	Single	Dual	Dual + modified	Multi-constriction
Read accuracy	<85%	~93–95%	~96–98%	~99% (Q20)	~99.3% (Q20+)	>99.9% (Q30)
Read length	Short-medium	Medium (N50 ~10 kb)	Ultra-long (>100 kb)	Ultra-long (>100 kb)	Ultra-long (>200 kb)	Ultra-long (>500 kb)
5mC detection	No	No	Limited	Limited	Yes (native, direct) [87]	Yes (6mA, 5hmC) [88]
Motor speed	~70 nt/s	~450 nt/s	~450 nt/s	~400 nt/s	~400 nt/s	Next-gen motor
Basecaller	Early Metrichor	Guppy (LSTM) [81]	Guppy/Bonito [81]	Guppy/Bonito	Dorado (GPU)	Dorado v5+
Key limitation	High error rate [80]	Homopolymer errors	Poor 5mC	Limited epigenetic detection	Higher library prep cost	Ongoing iterative optimization
Relevant to FSHD	No	Partial	Yes (D4Z4 spanning)	Yes (improved SV calling)	Yes (5mC at D4Z4, phasing)	Prospective

**Table 3:** Summary of published clinical data for PGT-M in FSHD.

Study	Cohort/Proband	Method	Embryo Classification	Clinical Outcome	Key Innovation
Xu et al. 2026 [37], SYSU	De novo mosaic FSHD1: 4/16/33 RUs, 4qA; male, 32y	Nanopore WGS + haplotype phasing + Sanger + NGS [37]	5/8 unaffected; 2 recomb. resolved by 3-tier	Healthy male live birth; OGM prenatal (17 + 33 RUs)	Block-based phasing; 224 kb recombination gap resolution; nanopolish + DSS
Hu et al. 2025 [38], CITIC-Xiangya	6 FSHD1 families; D4Z4 2–6 RUs, 4qA	MicroSeq + SNP linkage; Bionano OGM prenatal [38]	34 blastocysts; 18 unaffected (52.9%)	4 healthy live births; OGM confirmation	First successful FSHD1 PGT-M; MicroSeq linkage markers
Shani et al. 2023 [99], Israel	21 women from FSHD families	STR-based indirect linkage PGT-M [99]	268 embryos tested	17 healthy live births	Largest FSHD PGT-M case series reported

## Data Availability

No new data were created or analyzed in this study. Data sharing is not applicable to this article.

## References

[1] Deenen J.C., Arnts H., van der Maarel S.M., Padberg G.W., Verschuuren J.J., Bakker E., Weinreich S.S., Verbeek A.L., van Engelen B.G. (2014). Population-based incidence and prevalence of facioscapulohumeral dystrophy. Neurology.

[2] Deenen J.C., Horlings C.G., Verschuuren J.J., Verbeek A.L., van Engelen B.G. (2015). The Epidemiology of Neuromuscular Disorders: A Comprehensive Overview of the Literature. J. Neuromuscul. Dis..

[3] Wang Z., Qiu L., Lin M., Chen L., Zheng F., Lin L., Lin F., Ye Z., Lin X., He J. (2022). Prevalence and disease progression of genetically-confirmed facioscapulohumeral muscular dystrophy type 1 (FSHD1) in China between 2001 and 2020. Lancet Reg. Health West. Pac..

[4] Magdinier F. (2022). Joining mainstream research on Facioscapulohumeral Dystrophy: Disease prevalence in China. Lancet Reg. Health West. Pac..

[5] Tawil R., Kissel J.T., Heatwole C., Pandya S., Gronseth G., Benatar M. (2015). Evidence-based guideline summary: Evaluation, diagnosis, and management of facioscapulohumeral muscular dystrophy. Neurology.

[6] Wagner K.R. (2019). Facioscapulohumeral Muscular Dystrophies. Continuum.

[7] Mul K., Lassche S., Voermans N.C., Padberg G.W., Horlings C.G., van Engelen B.G. (2016). What’s in a name? The clinical features of facioscapulohumeral muscular dystrophy. Pract. Neurol..

[8] Ricci G., Mele F., Govi M., Ruggiero L., Sera F., Vercelli L., Bettio C., Santoro L., Mongini T., Villa L. (2020). Large genotype-phenotype study in carriers of D4Z4 borderline alleles provides guidance for facioscapulohumeral muscular dystrophy diagnosis. Sci. Rep..

[9] Ruggiero L., Mele F., Manganelli F., Bruzzese D., Ricci G., Vercelli L., Govi M., Vallarola A., Tripodi S., Villa L. (2020). Phenotypic variability among patients with D4Z4 reduced allele facioscapulohumeral muscular dystrophy. JAMA Netw. Open.

[10] Gabriëls J., Beckers M.C., Ding H., De Vriese A., Plaisance S., van der Maarel S.M., Padberg G.W., Frants R.R., Hewitt J.E., Collen D. (1999). Nucleotide sequence of the partially deleted D4Z4 locus in a patient with FSHD identifies a putative gene within each 3.3 kb element. Gene.

[11] Lemmers R.J., van der Vliet P.J., Klooster R., Sacconi S., Camaño P., Dauwerse J.G., Snider L., Straasheijm K.R., van Ommen G.J., Padberg G.W. (2010). A unifying genetic model for facioscapulohumeral muscular dystrophy. Science.

[12] Snider L., Geng L.N., Lemmers R.J.L.F., Kyba M., Ware C.B., Nelson A.M., Tawil R., Filippova G.N., van der Maarel S.M., Tapscott S.J. (2010). Facioscapulohumeral dystrophy: Incomplete suppression of a retrotransposed gene. PLoS Genet..

[13] Geng L.N., Yao Z., Snider L., Fong A.P., Cech J.N., Young J.M., van der Maarel S.M., Ruzzo W.L., Gentleman R.C., Tawil R. (2012). DUX4 activates germline genes, retroelements, and immune mediators: Implications for facioscapulohumeral dystrophy. Dev. Cell.

[14] van Overveld P.G., Lemmers R.J., Sandkuijl L.A., Enthoven L., Winokur S.T., Bakels F., Padberg G.W., Van Ommen G.-J.B., Frants R.R., van der Maarel S.M. (2003). Hypomethylation of D4Z4 in 4q-linked and non-4q-linked facioscapulohumeral muscular dystrophy. Nat. Genet..

[15] de Greef J.C., Wohlgemuth M., Chan O.A., Hansson K.B., Smeets D., Frants R.R., Weemaes C.M., Padberg G.W., van der Maarel S.M. (2007). Hypomethylation is restricted to the D4Z4 repeat array in phenotypic FSHD. Neurology.

[16] Zeng W., de Greef J.C., Chen Y.Y., Chien R., Kong X., Gregson H.C., Winokur S.T., Pyle A., Robertson K.D., Schmiesing J.A. (2009). Specific loss of histone H3 lysine 9 trimethylation and HP1γ/cohesin binding at D4Z4 repeats is associated with facioscapulohumeral dystrophy (FSHD). PLoS Genet..

[17] Cabianca D.S., Casa V., Bodega B., Xynos A., Ginelli E., Tanaka Y., Gabellini D. (2012). A long ncRNA links copy number variation to a polycomb/trithorax epigenetic switch in FSHD muscular dystrophy. Cell.

[18] Lemmers R.J., Tawil R., Petek L.M., Balog J., Block G.J., Santen G.W.E., Amell A.M., van der Vliet P.J., Almomani R., Straasheijm K.R. (2012). Digenic inheritance of an SMCHD1 mutation and an FSHD-permissive D4Z4 allele causes facioscapulohumeral muscular dystrophy type 2. Nat. Genet..

[19] van den Boogaard M.L., Lemmers R.J., Balog J., Wohlgemuth M., Auranen M., Mitsuhashi S., van der Vliet P.J., Straasheijm K.R., van den Akker R.F.P., Kriek M. (2016). Mutations in DNMT3B modify epigenetic repression of the D4Z4 repeat and the penetrance of facioscapulohumeral dystrophy. Am. J. Hum. Genet..

[20] Lemmers R.J., Goeman J.J., van der Vliet P.J., van Nieuwenhuizen M.P., Balog J., Vos-Versteeg M., Camano P., Arroyo M.A.R., Jerico I., Rogers M.T. (2015). Inter-individual differences in CpG methylation at D4Z4 correlate with clinical variability in FSHD1 and FSHD2. Hum. Mol. Genet..

[21] Yao Z., Snider L., Balog J., Lemmers R.J., van der Maarel S.M., Tawil R., Tapscott S.J. (2014). DUX4-induced gene expression is the major molecular signature in FSHD skeletal muscle. Hum. Mol. Genet..

[22] Whiddon J.L., Langford A.T., Wong C.J., Zhong J.W., Tapscott S.J. (2017). Conservation and innovation in the DUX4-family gene network. Nat. Genet..

[23] Hendrickson P.G., Doráis J.A., Grow E.J., Whiddon J.L., Lim J.-W., Wike C.L., Weaver B.D., Pflueger C., Emery B.R., Wilcox A.L. (2017). Conserved roles of mouse DUX and human DUX4 in activating cleavage-stage genes and MERVL/HERVL retrotransposons. Nat. Genet..

[24] Dixit M., Ansseau E., Tassin A., Winokur S., Shi R., Qian H., Sauvage S., Mattéotti C., van Acker A.M., Leo O. (2007). DUX4, a candidate gene of facioscapulohumeral muscular dystrophy, encodes a transcriptional activator of PITX1. Proc. Natl. Acad. Sci. USA.

[25] Rickard A.M., Petek L.M., Miller D.G. (2015). Endogenous DUX4 expression in FSHD myotubes is sufficient to cause cell death and disrupts RNA splicing and cell migration pathways. Hum. Mol. Genet..

[26] Shadle S.C., Zhong J.W., Campbell A.E., Conerly M.L., Jagannathan S., Wong C.-J., Morello T.D., van der Maarel S.M., Tapscott S.J. (2017). DUX4-induced dsRNA and MYC mRNA stabilization activate apoptotic pathways in human cell models of facioscapulohumeral dystrophy. PLoS Genet..

[27] Wallace L.M., Garwick S.E., Mei W., Belayew A., Coppee F., Ladner K.J., Guttridge D., Yang J., Harper S.Q. (2011). DUX4, a candidate gene for facioscapulohumeral muscular dystrophy, causes p53-dependent myopathy in vivo. Ann. Neurol..

[28] Bosnakovski D., Xu Z., Gang E.J., Galindo C.L., Liu M., Simsek T., Garner H.R., Agha-Mohammadi S., Tassin A., Coppée F. (2008). An isogenetic myoblast expression screen identifies DUX4-mediated FSHD-associated molecular pathologies. EMBO J..

[29] Vanderplanck C., Ansseau E., Charron S., Stricwant N., Tassin A., Laoudj-Chenivesse D., Wilton S.D., Coppée F., Belayew A. (2011). The FSHD atrophic myotube phenotype is caused by DUX4 expression. PLoS ONE.

[30] Banerji C.R., Panamarova M., Hebaishi H., White R.B., Relaix F., Severini S., Zammit P.S. (2017). PAX7 target genes are globally repressed in facioscapulohumeral muscular dystrophy skeletal muscle. Nat. Commun..

[31] Banerji C.R.S., Cammish P., Evangelista T., Zammit P.S., Straub V., Marini-Bettolo C. (2020). Facioscapulohumeral muscular dystrophy 1 patients participating in the UK FSHD registry can be subdivided into 4 patterns of self-reported symptoms. Neuromuscul. Disord..

[32] Butterfield R.J., Dunn D.M., Duval B., Moldt S., Weiss R.B. (2023). Deciphering D4Z4 CpG methylation gradients in fascioscapulohumeral muscular dystrophy using nanopore sequencing. Genome Res..

[33] Nurk S., Koren S., Rhie A., Rautiainen M., Bzikadze A.V., Mikheenko A., Vollger M.R., Altemose N., Uralsky L., Gershman A. (2022). The complete sequence of a human genome. Science.

[34] Giardina E., Camaño P., Burton-Jones S., Ravenscroft G., Henning F., Magdinier F., van der Stoep N., van der Vliet P.J., Bernard R., Tomaselli P.J. (2024). Best practice guidelines on genetic diagnostics of facioscapulohumeral muscular dystrophy: Update of the 2012 guidelines. Clin. Genet..

[35] Stence A.A., Thomason J.G., Pruessner J.A., Sompallae R.R., Snow A.N., Ma D., Moore S.A., Bossler A.D. (2021). Validation of Optical Genome Mapping for the Molecular Diagnosis of Facioscapulohumeral Muscular Dystrophy. J. Mol. Diagn..

[36] Guruju N.M., Jump V., Lemmers R.J., Van Der Maarel S., Liu R., Nallamilli B.R., Shenoy S., Chaubey A., Koppikar P., Rose R. (2023). Molecular diagnosis of facioscapulohumeral muscular dystrophy in patients clinically suspected of FSHD using optical genome mapping. Neurol. Genet..

[37] Xu Y., Wang J., Chen Y., Zeng Y., Wang Q., Fan H., Feng S., Huang X., Luo X., Pan J. (2026). Proband Nanopore Long-Read Genome Sequencing Facilitates Preimplantation Genetic Testing for Facioscapulohumeral Muscular Dystrophy. Neurol. Genet..

[38] Hu X., Wang W., Cheng D., Gu Y., Wan Z., Dai J., Zhang Y., Luo K., Li W., Zhang Q. (2025). Preimplantation genetic testing for facioscapulohumeral dystrophy caused by contractions of 4q35 D4Z4 repeats. Reprod. Biomed. Online.

[39] Carvalho F., Moutou C., Dimitriadou E., Dreesen J., Giménez C., Goossens V., Kakourou G., Vermeulen N., Zuccarello D., ESHRE PGT-M Working Group (2020). ESHRE PGT Consortium good practice recommendations for the detection of monogenic disorders. Hum. Reprod. Open.

[40] Pai E., Cameron E., McWilliams K. (2022). Review of recombination events observed in preimplantation genetic testing for monogenic disorders (PGT-M) over a 2 year period. Fertil. Steril..

[41] Deamer D., Akeson M., Branton D. (2016). Three decades of nanopore sequencing. Nat. Biotechnol..

[42] Jain M., Olsen H.E., Paten B., Akeson M. (2016). The Oxford Nanopore MinION: Delivery of nanopore sequencing to the genomics community. Genome Biol..

[43] Jain M., Koren S., Miga K.H., Quick J., Rand A.C., Sasani T.A., Tyson J.R., Beggs A.D., Dilthey A.T., Fiddes I.T. (2018). Nanopore sequencing and assembly of a human genome with ultra-long reads. Nat. Biotechnol..

[44] Wang Y., Zhao Y., Bollas A., Wang Y., Au K.F. (2021). Nanopore sequencing technology, bioinformatics and applications. Nat. Biotechnol..

[45] Huang M., Zhang Q., Jiao J., Shi J., Xu Y., Zhang C., Zhou R., Liu W., Liang Y., Chen H. (2024). Comprehensive genetic analysis of facioscapulohumeral muscular dystrophy by Nanopore long-read whole-genome sequencing. J. Transl. Med..

[46] Simpson J.T., Workman R.E., Zuzarte P.C., David M., Dursi L.J., Timp W. (2017). Detecting DNA cytosine methylation using nanopore sequencing. Nat. Methods.

[47] Rand A.C., Jain M., Eizenga J.M., Musselman-Brown A., Olsen H.E., Akeson M., Paten B. (2017). Mapping DNA methylation with high-throughput nanopore sequencing. Nat. Methods.

[48] Ni P., Huang N., Zhang Z., Wang D.-P., Liang F., Miao Y., Xiao C.-L., Luo F., Wang J. (2019). DeepSignal: Detecting DNA methylation state from Nanopore sequencing reads using deep-learning. Bioinformatics.

[49] Porubsky D., Ebert P., Audano P.A., Vollger M.R., Harvey W.T., Marijon P., Ebler J., Munson K.M., Sorensen M., Sulovari A. (2021). Fully phased human genome assembly without parental data using single-cell strand sequencing and long reads. Nat. Biotechnol..

[50] Kronenberg Z.N., Rhie A., Koren S., Concepcion G.T., Peluso P., Munson K.M., Porubsky D., Kuhn K., Mueller K.A., Low W.Y. (2021). Extended haplotype-phasing of long-read de novo genome assemblies using Hi-C. Nat. Commun..

[51] Aganezov S., Yan S.M., Soto D.C., Kirsche M., Zarate S., Avdeyev P., Taylor D.J., Shafin K., Shumate A., Xiao C. (2022). A complete reference genome improves analysis of human genetic variation. Science.

[52] Vollger M.R., Guitart X., Dishuck P.C., Mercuri L., Harvey W.T., Gershman A., Diekhans M., Sulovari A., Munson K.M., Lewis A.P. (2022). Segmental duplications and their variation in a complete human genome. Science.

[53] Altemose N., Logsdon G.A., Bzikadze A.V., Sidhwani P., Langley S.A., Caldas G.V., Hoyt S.J., Uralsky L., Ryabov F.D., Shew C.J. (2022). Complete genomic and epigenetic maps of human centromeres. Science.

[54] Hoyt S.J., Storer J.M., Hartley G.A., Grady P.G.S., Gershman A., de Lima L.G., Limouse C., Halabian R., Wojenski L., Rodriguez M. (2022). From telomere to telomere: The transcriptional and epigenetic state of human repeat elements. Science.

[55] Hewitt J.E., Lyle R., Clark L.N., Valleley E.M., Wright T.J., Wijmenga C., van Deutekom J.C., Francis F., Sharpe P.T., Hofker M. (1994). Analysis of the tandem repeat locus D4Z4 associated with facioscapulohumeral muscular dystrophy. Hum. Mol. Genet..

[56] Lyle R., Wright T.J., Clark L.N., Hewitt J.E. (1995). The FSHD-associated repeat, D4Z4, is a member of a dispersed family of homeobox-containing repeats, subsets of which are clustered on the short arms of the acrocentric chromosomes. Genomics.

[57] Wijmenga C., Hewitt J.E., Sandkuijl L.A., Clark L.N., Wright T.J., Dauwerse H.G., Gruter A.-M., Hofker M.H., Moerer P., Williamson R. (1992). Chromosome 4q DNA rearrangements associated with facioscapulohumeral muscular dystrophy. Nat. Genet..

[58] Winokur S.T., Chen Y.W., Masny P.S., Martin J.H., Ehmsen J.T., Tapscott S.J., van der Maarel S.M., Hayashi Y., Flanigan K.M. (2003). Expression profiling of FSHD muscle supports a defect in specific stages of myogenic differentiation. Hum. Mol. Genet..

[59] van der Maarel S.M., Tawil R., Tapscott S.J. (2011). Facioscapulohumeral muscular dystrophy and DUX4: Breaking the silence. Trends Mol. Med..

[60] Salsi V., Losi F., Pini S., Chiara M., Tupler R. (2026). Rethinking genomics of facioscapulohumeral muscular dystrophy in the telomere-to-telomere era: Pitfalls in the hidden landscape of D4Z4 repeats. Eur. J. Hum. Genet..

[61] Resnick R., Wong C.J., Hamm D.C., Bennett S.R., Skene P.J., Hake S.B., Henikoff S., van der Maarel S.M., Tapscott S.J. (2019). DUX4-induced histone variants H3.X and H3.Y mark DUX4 target genes for expression. Cell Rep..

[62] Tawil R., van der Maarel S.M., Tapscott S.J. (2014). Facioscapulohumeral dystrophy: The path to consensus on pathophysiology. Skelet. Muscle.

[63] Hamanaka K., Šikrová D., Mitsuhashi S., Masuda H., Sekiguchi Y., Sugiyama A., Shibuya K., Lemmers R.J., Goossens R., Ogawa M. (2020). Homozygous nonsense variant in LRIF1 associated with facioscapulohumeral muscular dystrophy. Neurology.

[64] Dion C., Roche S., Laberthonnière C., Broucqsault N., Mariot V., Xue S., Gurzau A.D., Nowak A., Gordon C.T., Gaillard M.-C. (2019). SMCHD1 is involved in de novo methylation of the DUX4-encoding D4Z4 macrosatellite. Nucleic Acids Res..

[65] Balog J., Thijssen P.E., Shadle S., Straasheijm K.R., Van Der Vliet P.J., Krom Y.D., van den Boogaard M.L., de Jong A., Lemmers R.J.L.F., Tawil R. (2015). Increased DUX4 expression during muscle differentiation correlates with decreased SMCHD1 protein levels at D4Z4. Epigenetics.

[66] Engal E., Sharma S., Aviel U., Taqatqa N., Juster S., Jaffe-Herman S., Bentata M., Geminder O., Gershon A., Lewis R. (2024). DNMT3B splicing dysregulation mediated by SMCHD1 loss contributes to DUX4 overexpression and FSHD pathogenesis. Sci. Adv..

[67] Bouwman L.F., den Hamer B., Verveer E.P., Lerink L.J.S., Krom Y.D., van der Maarel S.M., de Greef J.C. (2020). Dnmt3b regulates DUX4 expression in a tissue-dependent manner in transgenic D4Z4 mice. Skelet. Muscle.

[68] Sacconi S., Briand-Suleau A., Gros M., Baudoin C., Lemmers R.J., Rondeau S., Lagha N., Nigumann P., Cambieri C., Puma A. (2019). FSHD1 and FSHD2 form a disease continuum. Neurology.

[69] Jones T.I., Yan C., Sapp P.C., McKenna-Yasek D., Kang P.B., Quinn C., Salameh J.S., King O.D., Jones P.L. (2014). Identifying diagnostic DNA methylation profiles for facioscapulohumeral muscular dystrophy in blood and saliva using bisulfite sequencing. Clin. Epigenetics.

[70] de Greef J.C., Lemmers R.J., van Engelen B.G., Sacconi S., Venance S.L., Frants R.R., Tawil R., van der Maarel S.M. (2009). Common epigenetic changes of D4Z4 in contraction-dependent and contraction-independent FSHD. Hum. Mutat..

[71] Löwenstern T., Madritsch S., Horner D., Brait N., Lafci N.G., Schachner A., Bujalkova M.G., Kałużewski T., Szyld P., Hengstschläger M. (2026). DUCKS4: A comprehensive workflow for Nanopore sequencing analysis of facioscapulohumeral muscular dystrophy (FSHD). Hum. Genom..

[72] Vasale J., Boyar F., Jocson M., Sulcova V., Chan P., Liaquat K., Hoffman C., Meservey M., Chang I., Tsao D. (2015). Molecular combing compared to Southern blot for measuring D4Z4 contractions in FSHD. Neuromuscul. Disord..

[73] Nguyen K., Walrafen P., Bernard R., Attarian S., Chaix C., Vovan C., Renard E., Dufrane N., Pouget J., Vannier A. (2011). Molecular combing reveals allelic combinations in facioscapulohumeral dystrophy. Ann. Neurol..

[74] Nguyen K., Puppo F., Roche S., Gaillard M.-C., Chaix C., Lagarde A., Pierret M., Vovan C., Olschwang S., Salort-Campana E. (2017). Molecular combing reveals complex 4q35 rearrangements in Facioscapulohumeral dystrophy. Hum. Mutat..

[75] Park S.O., Kim M.J., Han G., Lee S., Hong Y.S., Kang H.R., Choi N., Lee J., Jeon M.S., Lee H.-S. (2023). Affect of chromosome recombination in target genes on PGT-M diagnosis using karyomapping analysis. Fertil. Steril..

[76] Spits C., Le Caignec C., De Rycke M., Van Haute L., Van Steirteghem A., Liebaers I., Sermon K. (2006). Whole-genome multiple displacement amplification from single cells. Nat. Protoc..

[77] Estévez-Gómez N., Prieto T., Tomás L., Alvariño P., Guillaumet-Adkins A., Heyn H., Prado-López S., Posada D. (2025). Differential performance of strategies for single-cell whole-genome amplification. Cell Rep. Methods.

[78] Kasianowicz J.J., Brandin E., Branton D., Deamer D.W. (1996). Characterization of individual polynucleotide molecules using a membrane channel. Proc. Natl. Acad. Sci. USA.

[79] Meller A., Nivon L., Brandin E., Golovchenko J., Branton D. (2000). Rapid nanopore discrimination between single polynucleotide molecules. Proc. Natl. Acad. Sci. USA.

[80] Laszlo A.H., Derrington I.M., Ross B.C., Brinkerhoff H., Adey A., Nova I.C., Craig J.M., Langford K.W., Samson J.M., Daza R. (2014). Decoding long nanopore sequencing reads of natural DNA. Nat. Biotechnol..

[81] Wick R.R., Judd L.M., Holt K.E. (2019). Performance of neural network basecalling tools for Oxford Nanopore sequencing. Genome Biol..

[82] Butler T.Z., Pavlenok M., Derrington I.M., Niederweis M., Gundlach J.H. (2008). Single-molecule DNA detection with an engineered MspA protein nanopore. Proc. Natl. Acad. Sci. USA.

[83] Manrao E.A., Derrington I.M., Laszlo A.H., Langford K.W., Hopper M.K., Gillgren N., Pavlenok M., Niederweis M., Gundlach J.H. (2012). Reading DNA at single-nucleotide resolution with a mutant MspA nanopore and phi29 DNA polymerase. Nat. Biotechnol..

[84] Cherf G.M., Lieberman K.R., Rashid H., Lam C.E., Karplus K., Akeson M. (2012). Automated forward and reverse ratcheting of DNA in a nanopore at 5-Å precision. Nat. Biotechnol..

[85] Sereika M., Kirkegaard R.H., Karst S.M., Michaelsen T.Y., Sørensen E.A., Wollenberg R.D., Albertsen M. (2022). Oxford Nanopore R10.4 long-read sequencing enables the generation of near-finished bacterial genomes from pure cultures and metagenomes without short-read or reference polishing. Nat. Methods.

[86] Kolmogorov M., Billingsley K.J., Mastoras M., Meredith M., Monlong J., Lorig-Roach R., Asri M., Jerez P.A., Malik L., Dewan R. (2023). Scalable Nanopore sequencing of human genomes provides a comprehensive view of haplotype-resolved variation and methylation. Nat. Methods.

[87] Sharim H., Grunwald A., Gabrieli T., Michaeli Y., Margalit S., Torchinsky D., Arielly R., Nifker G., Juhasz M., Gularek F. (2019). Long-read single-molecule maps of the functional methylome. Genome Res..

[88] Liu Y., Rosikiewicz W., Pan Z., Jillette N., Wang P., Taghbalout A., Foox J., Mason C., Carroll M., Cheng A. (2021). DNA methylation-calling tools for Oxford Nanopore sequencing: A survey and human epigenome-wide evaluation. Genome Biol..

[89] Goossens R., van den Boogaard M.L., Lemmers R.J., Balog J., van der Vliet P.J., Willemsen I.M., Schouten J., Maggio I., van der Stoep N., Hoeben R.C. (2019). Intronic SMCHD1 variants in FSHD: Testing the potential for CRISPR-Cas9 genome editing. J. Med. Genet..

[90] Xiao L.C., Semwal A., St John B., Zeglinski K., Su S., Lancaster J., Xue S., Reversade B., Ritchie M.E., Magdinier F. (2026). Complete genetic and epigenetic architecture of D4Z4 macrosatellites in FSHD, BAMS, and reference cohorts with D4Z4End2End. Genome Res..

[91] Eichler E.E., Clark R.A., She X. (2004). An assessment of the sequence gaps: Unfinished business in a finished human genome. Nat. Rev. Genet..

[92] Miller D.E., Sulovari A., Wang T., Loucks H., Hoekzema K., Munson K.M., Lewis A.P., Fuerte E.P.A., Paschal C.R., Walsh T. (2021). Targeted long-read sequencing identifies missing disease-causing variation. Am. J. Hum. Genet..

[93] Liao W.W., Asri M., Ebler J., Doerr D., Haukness M., Hickey G., Lu S., Lucas J.K., Monlong J., Abel H.J. (2023). A draft human pangenome reference. Nature.

[94] Miga K.H., Wang T. (2021). The Need for a Human Pangenome Reference Sequence. Annu. Rev. Genom. Hum. Genet..

[95] Practice Committee and Genetic Counseling Professional Group of the American Society for Reproductive Medicine, American Society for Reproductive Medicine (2023). Indications and management of preimplantation genetic testing for monogenic conditions: A committee opinion. Fertil. Steril..

[96] Tian Y., Li M., Yang J., Chen H., Lu D. (2024). Preimplantation genetic testing in the current era, a review. Arch. Gynecol. Obstet..

[97] Handyside A.H., Kontogianni E.H., Hardy K., Winston R.M. (1990). Pregnancies from biopsied human preimplantation embryos sexed by Y-specific DNA amplification. Nature.

[98] Chen S., Yin X., Zhang S., Xia J., Liu P., Xie P., Yan H., Liang X., Zhang J., Chen Y. (2021). Comprehensive preimplantation genetic testing by massively parallel sequencing. Hum. Reprod..

[99] Shani H., Feldman B., Inbar N., Reznik-Wolf H., Pras E. (2023). P557: Preimplantation genetic diagnosis for facioscapulohumeral muscular dystrophy. Genet. Med. Open.

[100] Alon I., Bussod I., Ravitsky V. (2024). Mapping ethical, legal, and social implications (ELSI) of preimplantation genetic testing (PGT). J. Assist. Reprod. Genet..

[101] Zhang J., Pastore L., Sarwana M., Klein S., Lobel M., Rubin L.R. (2022). Ethical and moral perspectives of individuals who considered/used preimplantation (embryo) genetic testing. J. Genet. Couns..

[102] Scotchman E., Chandler N.J., Mellis R., Chitty L.S. (2020). Non-invasive prenatal diagnosis and screening for monogenic disorders. Eur. J. Obstet. Gynecol. Reprod. Biol..

[103] Faust H., Duffek P., Hentschel J., Popp D. (2024). Evaluation of Automated Magnetic Bead-Based DNA Extraction for Detection of Short Tandem Repeat Expansions with Nanopore Sequencing. J. Clin. Lab. Anal..

[104] Senol Cali D., Kim J.S., Ghose S., Alkan C., Mutlu O. (2019). Nanopore sequencing technology and tools for genome assembly: Computational analysis of the current state, bottlenecks and future directions. Brief. Bioinform..

[105] Li H. (2018). Minimap2: Pairwise alignment for nucleotide sequences. Bioinformatics.

[106] Shafin K., Pesout T., Chang P.C., Nattestad M., Kolesnikov A., Goel S., Baid G., Kolmogorov M., Eizenga J.M., Miga K.H. (2021). Haplotype-aware variant calling with PEPPER-Margin-DeepVariant enables high accuracy in nanopore long-reads. Nat. Methods.

[107] De Coster W., Weissensteiner M.H., Sedlazeck F.J. (2021). Towards population-scale long-read sequencing. Nat. Rev. Genet..

[108] Ahsan M.U., Liu Q., Perdomo J.E., Fang L., Wang K. (2023). A survey of algorithms for the detection of genomic structural variants from long-read sequencing data. Nat. Methods.

[109] Kovaka S., Fan Y., Ni B., Timp W., Schatz M.C. (2021). Targeted nanopore sequencing by real-time mapping of raw electrical signal with UNCALLED. Nat. Biotechnol..

[110] Payne A., Holmes N., Clarke T., Munro R., Debebe B.J., Loose M. (2021). Readfish enables targeted nanopore sequencing of gigabase-sized genomes. Nat. Biotechnol..

[111] Gilpatrick T., Lee I., Graham J.E., Raimondeau E., Bowen R., Heron A., Downs B., Sukumar S., Sedlazeck F.J., Timp W. (2020). Targeted nanopore sequencing with Cas9-guided adapter ligation. Nat. Biotechnol..

[112] Stangl C., de Blank S., Renkens I., Westera L., Verbeek T., Valle-Inclan J.E., González R.C., Henssen A.G., van Roosmalen M.J., Stam R.W. (2020). Partner independent fusion gene detection by multiplexed CRISPR-Cas9 enrichment and long read nanopore sequencing. Nat. Commun..

[113] Garalde D.R., Snell E.A., Jachimowicz D., Sipos B., Lloyd J.H., Bruce M., Pantic N., Admassu T., James P., Warland A. (2018). Highly parallel direct RNA sequencing on an array of nanopores. Nat. Methods.

[114] Workman R.E., Tang A.D., Tang P.S., Jain M., Tyson J.R., Razaghi R., Zuzarte P.C., Gilpatrick T., Payne A., Quick J. (2019). Nanopore native RNA sequencing of a human poly(A) transcriptome. Nat. Methods.

[115] Leger A., Amaral P.P., Pandolfini L., Capitanchik C., Capraro F., Miano V., Migliori V., Toolan-Kerr P., Sideri T., Enright A.J. (2021). RNA modifications detection by comparative Nanopore direct RNA sequencing. Nat. Commun..

[116] Mitsuhashi S., Nakagawa S., Sasaki-Honda M., Sakurai H., Frith M.C., Mitsuhashi H. (2021). Nanopore direct RNA sequencing detects DUX4-activated repeats and isoforms in human muscle cells. Hum. Mol. Genet..

[117] Jain M., Abu-Shumays R., Olsen H.E., Akeson M. (2022). Advances in nanopore direct RNA sequencing. Nat. Methods.

[118] Tawil R., Wagner K.R., Hamel J.I., Leung D.G., Statland J.M., Wang L.H., Genge A., Sacconi S., Lochmüller H., Reyes-Leiva D. (2024). Safety and efficacy of losmapimod in facioscapulohumeral muscular dystrophy (ReDUX4): A randomised, double-blind, placebo-controlled phase 2b trial. Lancet Neurol..

[119] Mellion M.L., Ronco L., Berends C.L., Pagan L., Brooks S., van Esdonk M.J., van Brummelen E.M.J., Odueyungbo A., Thompson L.A., Hage M. (2021). Phase 1 clinical trial of losmapimod in facioscapulohumeral dystrophy: Safety, tolerability, pharmacokinetics, and target engagement. Br. J. Clin. Pharmacol..

[120] Oliva J., Galasinski S., Richey A., Campbell A.E., Meyers M.J., Modi N., Zhong J.W., Tawil R., Tapscott S.J., Sverdrup F.M. (2019). Clinically advanced p38 inhibitors suppress DUX4 expression in cellular and animal models of facioscapulohumeral muscular dystrophy. J. Pharmacol. Exp. Ther..

[121] Ansseau E., Vanderplanck C., Wauters A., Harper S.Q., Coppée F., Belayew A. (2017). Antisense oligonucleotides used to target the DUX4 mRNA as therapeutic approaches in facioscapulohumeral muscular dystrophy (FSHD). Genes.

[122] Marsollier A.C., Ciszewski L., Mariot V., Popplewell L., Voit T., Dickson G., Dumonceaux J. (2016). Antisense targeting of 3′ end elements involved in DUX4 mRNA processing is an efficient therapeutic strategy for facioscapulohumeral dystrophy: A new gene-silencing approach. Hum. Mol. Genet..

[123] Wallace L.M., Liu J., Domire J.S., Garwick-Coppens S.E., Guckes S.M., Mendell J.R., Flanigan K.M., Harper S.Q. (2012). RNA interference inhibits DUX4-induced muscle toxicity in vivo: Implications for a targeted FSHD therapy. Mol. Ther..

[124] Campbell A.E., Oliva J., Yates M.P., Zhong J.W., Shadle S.C., Snider L., Singh N., Tai S., Hiramuki Y., Tawil R. (2017). BET bromodomain inhibitors and agonists of the beta-2 adrenergic receptor identified in screens for compounds that inhibit DUX4 expression in FSHD muscle cells. Skelet. Muscle.

[125] Kowaljow V., Marcowycz A., Ansseau E., Conde C.B., Sauvage S., Mattéotti C., Arias C., Corona E.D., Nuñez N.G., Leo O. (2007). The DUX4 gene at the FSHD1A locus encodes a pro-apoptotic protein. Neuromuscul. Disord..

[126] Wang L.H., Tawil R. (2016). Facioscapulohumeral Dystrophy. Curr. Neurol. Neurosci. Rep..

[127] Choi S.H., Gearhart M.D., Cui Z., Bosnakovski D., Kim M., Schennum N., Kyba M. (2016). DUX4 recruits p300/CBP through its C-terminus and induces global H3K27 acetylation changes. Nucleic Acids Res..

[128] Jagannathan S., Shadle S.C., Resnick R., Snider L., Tawil R.N., van der Maarel S.M., Bradley R.K., Tapscott S.J. (2016). Model systems of DUX4 expression recapitulate the transcriptional profile of FSHD cells. Hum. Mol. Genet..

[129] Block G.J., Narayanan D., Amell A.M., Petek L.M., Davidson K.C., Bird T.D., Tawil R., Moon R.T., Miller D.G. (2013). Wnt/β-catenin signaling suppresses DUX4 expression and prevents apoptosis of FSHD muscle cells. Hum. Mol. Genet..

[130] Bodega B., Ramirez G.D., Grasser F., Cheli S., Brunelli S., Mora M., Meneveri R., Marozzi A., Mueller S., Battaglioli E. (2009). Remodeling of the chromatin structure of the facioscapulohumeral muscular dystrophy (FSHD) locus and upregulation of FSHD-related gene 1 (FRG1) expression during human myogenic differentiation. BMC Biol..

[131] Calandra P., Cascino I., Lemmers R.J., Galluzzi G., Teveroni E., Monforte M., Tasca G., Ricci E., Moretti F., van der Maarel S.M. (2016). Allele-specific DNA hypomethylation characterises FSHD1 and FSHD2. J. Med. Genet..

[132] Himeda C.L., Jones T.I., Jones P.L. (2016). CRISPR/dCas9-mediated transcriptional inhibition ameliorates the epigenetic dysregulation at D4Z4 and represses DUX4-fl in FSH muscular dystrophy. Mol. Ther..

[133] Gaillard M.C., Broucqsault N., Morere J., Laberthonnière C., Dion C., Badja C., Roche S., Nguyen K., Magdinier F., Robin J.D. (2019). Analysis of the 4q35 chromatin organization reveals distinct long-range interactions in patients affected with Facio-Scapulo-Humeral Dystrophy. Sci. Rep..

[134] Sacconi S., Camaño P., de Greef J.C., Lemmers R.J.L.F., Salviati L., Boileau P., Arregui A.L.d.M., van der Maarel S.M., Desnuelle C. (2012). Patients with a phenotype consistent with facioscapulohumeral muscular dystrophy display genetic and epigenetic heterogeneity. J. Med. Genet..

[135] Lemmers R.J., van der Vliet P.J., Balog J., Goeman J.J., Arindrarto W., Krom Y.D., Straasheijm K.R., Debipersad R.D., Özel G., Sowden J. (2018). Deep characterization of a common D4Z4 variant identifies biallelic DUX4 expression as a modifier for disease penetrance in FSHD2. Eur. J. Hum. Genet..

[136] Greco A., Goossens R., van Engelen B., van der Maarel S. (2020). Consequences of epigenetic derepression in facioscapulohumeral muscular dystrophy. Clin. Genet..

